# Persistence and metabolism of the diamide insecticide cyantraniliprole in tomato plants

**DOI:** 10.1038/s41598-021-00970-8

**Published:** 2021-11-03

**Authors:** Khang Huynh, Elizabeth Leonard, Juang-Horng Chong, Cristi Palmer, Nishanth Tharayil

**Affiliations:** 1 Department of Plant and Environmental Sciences, Clemson University, Clemson, SC 29634 USA; 2 Department of Plant and Environmental Sciences, Pee Dee Research and Education Center, Clemson University, Florence, SC 29506 USA; 3 Rutgers, The State University of New Jersey, IR-4 Project, New Brunswick, NJ 08901 USA

**Keywords:** Environmental chemistry, Metabolomics, Mass spectrometry

## Abstract

Plant uptake and metabolism of pesticides are complex and dynamic processes, which contribute to the overall toxicity of the pesticides. We investigated the metabolic fate of cyantraniliprole, a new diamide class of insecticide, during various growth stages of tomato. Cyantraniliprole was the major residue in leaves, flowers, and fruits, with the relative metabolite-to-parent ratios maintained at < 10% up to 28 days after treatment (DAT). Mature leaves contained consistently higher residues of cyantraniliprole than young leaves throughout the study. Flowers contained the highest cyantraniliprole residues up to 21 DAT, then gradually decreased. Immature green fruits had the highest cyantraniliprole residues (5.3 ± 0.7 ng/g; 42 DAT), and decreased toward red ripening stages (1.4 ± 0.2 ng/g; 84 DAT). Metabolism of cyantraniliprole primarily occurred in the foliage, where 21 metabolites were tentatively identified. Flowers and fruits contained 14 and four of these metabolites, respectively. Major transformation pathways were characterized by ring closure, followed by *N*-demethylation, and glycosylation. Additionally, plant metabolism of cyantraniliprole was also associated with several minor phase-I, phase-II, and breakdown metabolites. The occurrence of these metabolites in plants varied as a function of tissue types and their developmental stages. Our study highlights a tissue-specific biotransformation and accumulation of metabolites of cyantraniliprole in tomato.

## Introduction

Pesticides are the mainstay of modern agriculture, enabling growers to reduce pest infestation in crops. However, widespread pesticide use has also prompted persistent public concerns over the adverse effects of residues on non-target organisms and human health. Partly driven by this concern, over the past decades, the amount of pesticides used in the United States has been reduced by approximately 40%, shifting toward pyrethroids and neonicotinoids, which are less toxic to birds and mammals^1^. Unfortunately, this shifting trend has exerted significant threats to some plant species and invertebrates (e.g., pollinators)^1^. Besides, incidences of resistance to pyrethroids and neonicotinoids have also been reported worldwide^2–7^, necessitating the introduction of new chemistries for pest management.

In this context, recently introduced diamide insecticides have been considered the most promising alternative, in large part due to their lower toxicity to beneficial arthropods, mammals, and pollinators^8–10^. Diamide insecticides target insects’ ryanodine receptors and trigger uncontrolled release of internal calcium stores; consequently, the exposed insects suffer feeding cessation, lethargy, muscle paralysis, and eventually death^11^. Anthranilic diamides, such as cyantraniliprole and chlorantraniliprole, can be delivered via seed treatment, soil, or foliar applications to manage a broad-spectrum of chewing and sap sucking pests on several plant species^12–14^. Recent studies have suggested that soil application or seed treatment of cyantraniliprole and chlorantraniliprole could provide greater efficacy than foliar spray^15^ or equivalent level of pest protection compared to neonicotinoid seed treatment^8^, thereby reducing negative impacts on non-target species and environmental health concerns, and a potentially long residual effect post-application^8,15^. Nevertheless, their persistence in soils may result in a prolonged exposure of the treated crops and pests to the insecticides, potentially leading to increased bioaccumulation of the residues inside plant tissues and insecticide resistance^16^. Although the long residual efficacy of anthranilic diamides against pest infestation has been well-studied^8,10,15^, the metabolic pathways and distribution patterns of the residues and metabolites in crops during this prolonged exposure is less known. Furthermore, root uptake and transport of systemic insecticides largely depend on their physiochemical properties, leading to different distribution patterns and metabolite concentrations inside plant tissues compared to foliar application^17,18^. Plant detoxification systems are also influenced by environmental and physiological factors during the plant’s life cycle^19^, resulting in ontogeny-/tissue-dependent metabolite profiles. It has been shown that, in addition to the parent compounds, some insecticide metabolites may also retain insecticidal activity and could contribute to the overall toxicity of the residues^20–23^. Therefore, a comprehensive understanding of the metabolic fate and behavior of insecticides in plant tissues is of great importance for informing risk assessment.

In the present study, the uptake and distribution of soil-applied cyantraniliprole in different tissues of tomato plants (*Solanum lycopersicum* L.) and the associated transformation pathways were investigated. Tomato is an economically important crop around the world^24^ and represents a fruiting vegetable in which cyantraniliprole is effective against several insect pests (e.g., armyworm, aphids, hornworm, leafminer, loopers, psyllids, thrips, and whitefly)^25,26^. Targeted and untargeted metabolomics using high resolution mass-spectral information was used to identify the metabolite candidates, as well as variations in the metabolite profiles in the foliage, flowers, and fruits across different developmental stages of the tomato plants.

## Results and discussion

### Residues of cyantraniliprole in xylem saps and plant tissues

**Figure 1:**
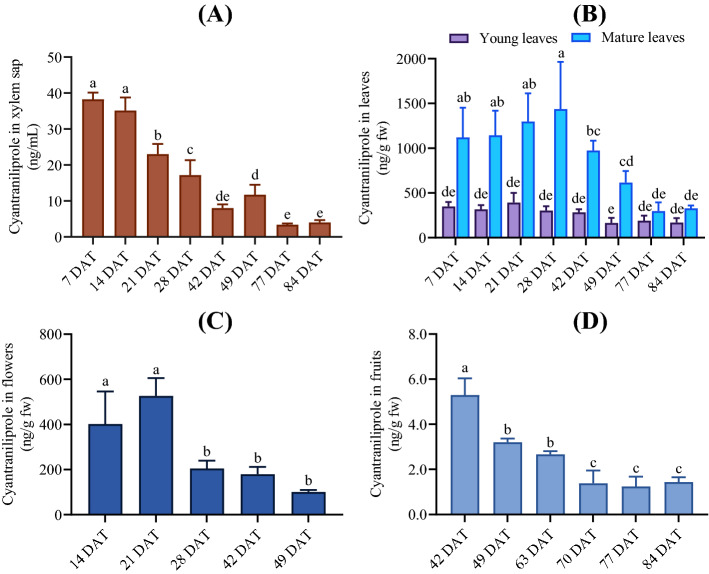
Concentrations of cyantraniliprole in xylem sap (**A**), leaves (**B**), flowers (**C**), and fruits (**D**) of tomato plants at different sampling times after treatment with Mainspring^®^ GNL. The values represent the means ± standard deviation (*n* = 5). Columns within the same graph topped by different letters are significantly different from each other (*p* < 0.05).

No phytotoxicity was observed for the plants treated with cyantraniliprole throughout the study, and the treated plants showed equivalent growth and development compared to the control plants. Following soil application, cyantraniliprole was quickly absorbed by roots and transported acropetally via the evapotranspiration stream in xylem. The highest concentrations of cyantraniliprole in the xylem sap were observed at 7 and 14 DAT (38.3 ± 1.9 and 35.1 ± 3.7 ng/mL, respectively) and decreased gradually in subsequent samples, reaching 4.0 ± 0.7 ng/mL at 84 DAT (Fig. 1A). Cyantraniliprole was also detected in all plant tissues at all sampling intervals up to fruit maturity, with residue concentrations declining in the order of foliage > flowers > fruits (Fig. 1). In the foliage, there was significant interaction between leaf age and sampling interval in determining residue concentrations (*p* < 0.001). The highest concentrations were found in mature leaves, increased from 1120.3 ± 332.3 ng/g at 7 DAT to 1437.8 ± 525.8 ng/g at 28 DAT. During the same period (7–28 DAT), the concentrations of cyantraniliprole accumulated in young (apical) leaves were significantly lower than those in mature leaves (*p* < 0.001), but similar among young leaves sampled at different times (ranged from 303.0 ± 47.3 at 28 DAT to 392.4 ± 108.1 ng/g at 21 DAT). After 28 DAT, residue concentrations in mature leaves decreased considerably, but those in young leaves decreased only slightly as sampling progressed, thus reducing difference between young and mature leaves. At 84 DAT, residues in mature and young leaves were 327.1 ± 32.1 ng/g and 169.3 ± 49.8 ng/g, respectively. Flowers exhibited peak residue concentration at 21 DAT (526.3 ± 79.4 ng/g) but decreased rapidly, reaching 101.3 ± 8.4 ng/g at the last flower harvest (49 DAT). Concentrations of cyantraniliprole in flowers were similar to those in young leaves (*p* = 0.67). In fruits, cyantraniliprole residue decreased with sampling intervals from its highest concentration at the immature green stage (42 DAT; 5.3 ± 0.7 ng/g) to its lowest at the red ripening stage (84 DAT; 1.4 ± 0.2 ng/g).

Since phloem mobility is absent, xylem flux was the predominant driving force that distributed cyantraniliprole throughout the plants following root uptake^15,25,27^. Accordingly, the higher residue concentrations in mature leaves compared with other tissues may be explained by the relative position and flow velocity for each respective plant tissues within the evapotranspiration stream^25^. Young leaves and flowers located in the positions of the farthest upward-transport along the xylem flux from soil pore water to plant apex. On the other hand, appreciable discrepancies in relative surface area of tomato leaves (6 m^2^/kg) and fruits (0.16 m^2^/kg)^25^ suggested that the xylem flux for leaves to be significantly larger than for fruits. Tomato leaves transpire water through stomata on the surfaces, creating a driving force for water and soluble compounds flow into leaf tissues, as opposed to fruits, which lack stomata^28,29^. Consequently, significantly lower concentrations of cyantraniliprole were found in the fruits compared to young leaves and mature leaves at all sampling intervals (*p* < 0.001). The reduction in cyantraniliprole concentrations in all plant tissues with the progression of sampling time can be explained by growth dilution effect and plant metabolism in the respective tissues, as well as a lower cyantraniliprole uptake from the growing medium as supported by the change in concentrations of the insecticide in xylem sap.

### Metabolic fate of cyantraniliprole in tomato plants

Due to the lack of reference standards, the metabolites of cyantraniliprole identified in the present study were confirmed at various confidence levels based on the framework proposed by Schymanski et al.^30^. The identification was based on the following criteria: (1) mass error < 5 ppm for the calculated and observed accurate *m/z*; (2) Cl/Br isotope patterns; (3) the proposed formulas obey the nitrogen rule^31^; and (4) the proposed chemical structures can be fully explained by MS^2^ fragment ions. Those metabolites with inadequate MS^2^ characteristic fragments were assigned to the lowest confidence level (level 5).

In this study, a total of 21 metabolites were tentatively identified in different tissues of the tomato plants treated with cyantraniliprole, including those that were reported in the registration documents^32^ as well as several new metabolites (Table 1). For example, the metabolite at *m/z* 455.0022 was detected in all treatments at a retention time of 11.04 min. The proposed elemental composition for this metabolite is C_19_H_12_BrClN_6_O, with a mass error of 1.1 ppm. Its isotope pattern was consistent with a molecule containing one bromine and one chlorine atom. Based on its accurate *m/z* and proposed formula, this metabolite was proposed as IN-J9Z38, a major metabolite of cyantraniliprole reported in previous studies^32–34^. MS^2^ fragment ions were subsequently used to confirm the chemical structure (Fig. S2). Similarly, the accurate masses of metabolite TP651 (*m/z* 651.0608 at RT = 6.53 min and *m/z* 651.0616 at RT = 7.21 min) allowed the prediction of the chemical formula C_25_H_24_BrClN_6_O_8_ with a mass error of 1.2 and 2.5 ppm, respectively. MS^2^ fragmentation revealed identical product ions at *m/z* 489.00, which is the characteristic molecular ion of hydroxylated cyantraniliprole. Addition of an anhydroglucose moiety (162.05 Da) suggested the formation of glycosylated metabolites (Figs. S17 and S18). The same approach was used for identification and confirmation of the remaining metabolites (Table 1). Out of 21 metabolites tentatively identified, 11 compounds were phase-I transformation products, six compounds were phase-II conjugates, and four compounds were breakdown products. In general, cyantraniliprole lacks the functional groups suitable for direct phase-II conjugation reactions (e.g., OH–, NH_2_–), which requires activation via phase-I reactions. This may explain the predominant presence of phase-I transformation products in the metabolite profile. Details of the proposed chemical structures and high-resolution mass spectra of all metabolites can be found in the Supporting Information (Figs. S2–S22).

**Table 1:** Mass-spectral information and proposed chemical formulas of cyantraniliprole-transformation products identified by Compound Discoverer 3.1 and Mass Frontier 8.0 software.

Denotation	Chemical formula	RT (min)^a^	Calcd *m/z*^b^	Obsd *m/z*^c^	Error (ppm)	Fragment ions (*m/z*)^d^	Leaves^e^	Flowers^f^	Fruits^g^	Level^h^
Cyantraniliprole	C_19_H_14_BrClN_6_O_2_	9.31	473.0123	473.0126	0.6	284, 442	+	+	+	1
IN-J9Z38	C_19_H_12_BrClN_6_O	11.04	455.0017	455.0022	1.1	350, 361, 375, 398, 419	+	+	−	2b
IN-RNU71	C_19_H_13_BrN_6_O_2_	8.15	437.0356	437.0361	1.1	301, 327, 406	+	+	−	2b
IN-HGW87	C_18_H_12_BrClN_6_O_2_	8.30	458.9966	458.9972	1.3	284, 442	+	−	−	2b
IN-JSE76	C_19_H_15_BrClN_5_O_4_	8.40	492.0069	492.0077	1.6	284, 461	+	−	−	2b
IN-JCZ38	C_19_H_16_BrClN_6_O_3_	7.59	491.0228	491.0235	1.3	284, 460	+	+	−	2b
IN-MLA84	C_18_H_10_BrClN_6_O	10.27	440.9861	440.9866	1.1	270, 284, 312, 361, 405	+	+	+	2b
IN-MYX98	C_19_H_14_BrClN_6_O_3_	8.44	489.0072	489.0078	1.2	442, 471	+	+	−	2b
IN-DBC80	C_9_H_5_BrClN_3_O_2_	7.77	301.9326	301.9328	0.7	258, 284	+	+	+	2b
IN-M2G98	C_9_H_6_BrClN_4_O	7.32	300.9486	300.9486	0.0	n.a	+	+	−	5
TP315	C_10_H_8_BrClN_4_O	7.80	314.9643	314.9644	0.3	284	+	+	−	3
TP316	C_10_H_7_BrClN_3_O_2_	10.11	315.9483	315.9486	1.0	n.a	+	+	+	5
TP363	C_18_H_11_ClN_6_O	8.99	363.0756	363.0760	1.1	206, 234, 270, 327	+	+	−	3
TP405	C_18_H_9_BrN_6_O	11.51	405.0094	405.0100	1.5	298, 326	+	−	−	3
TP423	C_18_H_11_BrN_6_O_2_	7.79	423.0200	423.0207	1.7	262, 289, 327, 406	+	+	−	3
TP441	C_18_H_10_BrClN_6_O	8.42	440.9861	440.9865	0.9	284, 405	+	−	−	3
TP577	C_22_H_18_BrClN_6_O_4_S	8.67	577.0055	577.0063	1.4	n.a	+	+	−	5
TP619	C_24_H_20_BrClN_6_O_7_	6.95	619.0338	619.0347	1.5	403, 439, 457	+	+	+	3
TP633	C_25_H_22_BrClN_6_O_7_	7.67	633.0495	633.0503	1.3	471	+	−	−	3
TP651a	C_25_H_24_BrClN_6_O_8_	6.53	651.0600	651.0608	1.2	284, 458, 471, 489	+	+	−	3
TP651b	C_25_H_24_BrClN_6_O_8_	7.21	651.0600	651.0616	2.5	442, 471, 489	+	−	−	3
TP654	C_25_H_25_BrClN_5_O_9_	7.20	654.0597	654.0608	1.7	284, 445, 461, 474, 623	+	−	−	3

n.a: not available, (+): detected, (−): not detected.

^a^Retention time of cyantraniliprole and its metabolites on the UPLC-Orbitrap-MS system.

^b^The accurate calculated masses (calcd *m/z*) were obtained with Chemsketch software, version 2019.1.2 (ACD/Laboratories, Toronto, ON).

^c^The observed masses (obsd *m/z*) were obtained from a high-resolution MS (Thermo Orbitrap Fusion™ Tribrid™).

^d^The fragments ions acquired using data-dependent MS^2^ fragmentation in CID mode of the UPLC-Orbitrap-MS.

^e,f,g^Detected in leaves, flowers, and fruits, respectively.

^h^According to Schymanski et al.^30^ Level 1: reference standard, HR-MS, MS/MS, RT confirmed; Level 2b: HR-MS, characteristic fragmentation patterns observed; metabolites previously reported^32^; Level 3: HR-MS, characteristic fragmentation patterns observed, alternative structures (e.g., glycosylation positions) might be possible; Level 5: exact mass of interest.

**Figure 2:**
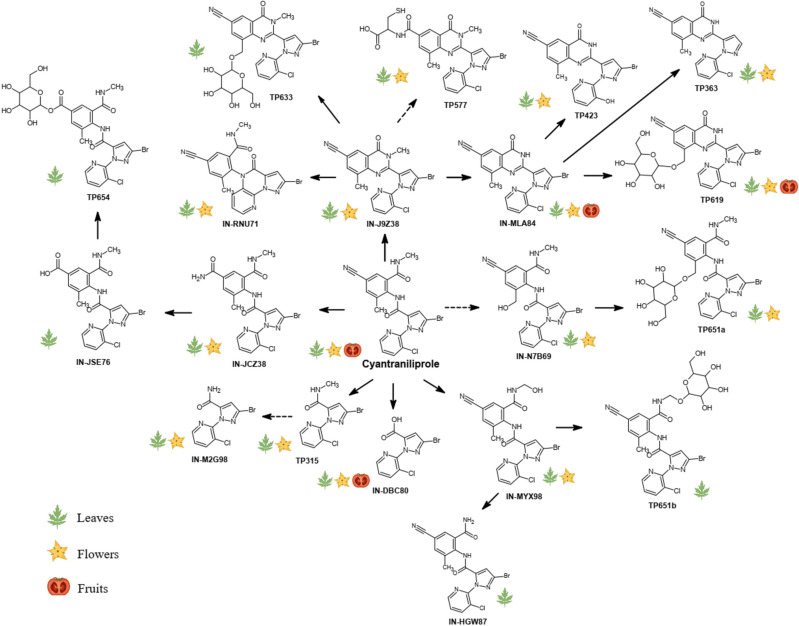
Proposed transformation pathways of cyantraniliprole in tomato plant tissues. Dashed arrows represent the pathways to the metabolites that have previously been reported^32,35,36^, or to the new metabolites identified in this study, in which their presence could not be unambiguously confirmed due to insufficient characteristic product ions acquired during MS^2^ fragmentation. The icons depicting leaves, flowers, and fruits indicate the occurrence of cyantraniliprole and its metabolites in the respective tissues.

Based on our results and previous reports^32,35,36^, the metabolic pathways involved in the transformation of cyantraniliprole in tomato plants are presented in Fig. 2. We observed that ring closure, followed by *N*-demethylation were the prominent phase-I transformation pathways of cyantraniliprole in tomato plants, leading to the formation of IN-J9Z38 and IN-MLA84, respectively. The metabolite IN-J9Z38 then served as a key intermediate for several subsequent biotransformation reactions. Both IN-J9Z38 and IN-MLA84 were readily glycosylated to form the newly identified metabolites TP633 and TP619, respectively. Additionally, IN-J9Z38 and IN-MLA84 were dechlorinated and hydroxylated on the pyridine ring to form IN-RNU71 and TP423, respectively. IN-MLA84 could also be debrominated to form TP363. Oxidation at the cyano group of IN-J9Z38, followed by conjugation with the amino acid cysteine gave rise to the metabolite TP577. On the other hand, cyantraniliprole also underwent hydroxylation at the *N*-methyl group to form IN-MYX98 which was subsequently glycosylated to TP651. There were two signals with similar accurate *m/z* 651.061 ± 0.001 Da, in which the MS^2^ fragmentation gave rise to identical product ions at *m/z* 489.008 ± 0.001 Da, which is the characteristic molecular ion of hydroxylated cyantraniliprole (Table 1). The isomeric signals of TP651 were denoted as TP651a (Fig. S17) and TP651b (Fig. S18), respectively. Although only IN-MYX98 was detected in this study, the second precursor hydroxylated metabolite was presumably IN-N7B69, which has commonly been observed in other plant species^32^. The metabolite IN-MYX98 could also be *N*-dealkylated to form IN-HGW87, the free amide of parent cyantraniliprole. In another pathway, the cyano group of cyantraniliprole underwent varying degrees of oxidation to amide (IN-JCZ38) and carboxylic acid (IN-JSE76) moieties. Glucose conjugation of IN-JSE76 was also observed, which was denoted as TP654. The breakdown metabolites IN-DBC80, IN-M2G98, and TP315, formed by cleavage of the carboxamide bridge between the phenyl and pyridine rings, could be from the parent cyantraniliprole or metabolites.

### Distribution of cyantraniliprole metabolites in different plant tissues

The observed cyantraniliprole metabolites in tomato tissues could be the products of plant metabolism or soil degradants that were subsequently taken up. However, these metabolites were undetectable at all sampling intervals in xylem saps, suggesting that root-to-shoot translocation of cyantraniliprole metabolites was negligible, if any, and that transformation of cyantraniliprole in tomato plants primarily occurred in the above-ground plant tissues. Compared to young leaves, mature leaves were the major reservoir of cyantraniliprole metabolites. Cyantraniliprole metabolites were also detected in flower and fruit samples (Fig. 2); however, their occurrence in these tissues was quantitively lower than that in the foliage.

**Figure 3:**
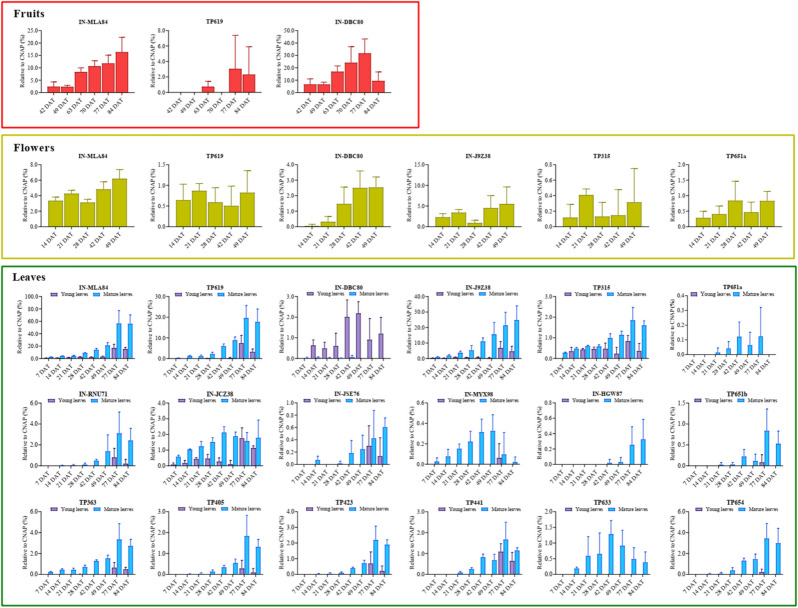
Relative abundances of cyantraniliprole metabolites detected in tomato leaves, flowers, and fruits throughout the experiments. For flowers, the metabolites that were only detectable in the concentrated extracts as described in the Materials and Methods, including IN-M2G98, IN-RNU71, IN-MYX98, IN-JCZ38, TP363, and TP423, are not presented due to their negligible abundance compared to parent cyantraniliprole. Also, other metabolites confirmed at the lowest confidence level (level 5, Table 1), including IN-M2G98, TP316, and TP577, are not presented for all plant tissues. The values represent the means ± standard deviation (*n* = 5).

In order to visualize the variations of the metabolites in different tissues and sampling intervals, the intensity of each metabolite was normalized to that of cyantraniliprole at the corresponding sampling interval and presented as the metabolite-to-parent (M/P) ratio. Relative abundances of the tentatively identified cyantraniliprole metabolites in young and mature leaves, flowers, and fruits are shown in Fig. 3. The corresponding peak area data prior to normalization can be found in the Supporting Information (Fig. S25). In the foliage, the M/P ratios of all detected metabolites were < 10% up to 28 DAT, indicating that cyantraniliprole was relatively recalcitrant to metabolism. IN-J9Z38, IN-MLA84, and TP619 were found to be the major metabolites in mature leaves, and their relative abundances increased throughout the sampling period. The highest M/P ratios of IN-J9Z38, IN-MLA84 and TP619 were approximately 24.9, 56.1, and 17.7% at 84 DAT, respectively. The increase of these major metabolites in mature leaves could be partly explained by the enhanced biotransformation of cyantraniliprole, as well as depleted store of cyantraniliprole in the growth medium toward the conclusion of the experiment, leading to significant reduction of the parent insecticide residues in mature leaves (Fig. 1B). In young leaves, the M/P ratios of both IN-J9Z38 and TP619 were < 10% across all sampling intervals, while those of IN-MLA84 was approximately 15.2% at 84 DAT (Fig. 3), likely attributed to lower enzyme activity compared to mature leaves^37^. Both IN-J9Z38 and IN-MLA84 were also present as major residues in studies with rotational crops (e.g., wheat forage, hay, straw, and soybean foliage), while primary crop studies reported the formation of several low abundance metabolites^32^. We observed the occurrence of TP619, tentatively identified as a glycosylated conjugate of IN-MLA84, as a major metabolite of cyantraniliprole in tomato plants for the first time in this study. Apart from the foliage, IN-J9Z38, IN-MLA84, and TP619 were also detected in the flowers sampled at different times, with the highest M/P ratios at approximately 5.5, 6.2, and 0.9%, respectively (Fig. 3). In the fruits, IN-J9Z38 was undetectable at any developmental stages, whereas IN-MLA84 was found to be a major metabolite, with the M/P ratio increased from approximately 2.4% at the immature green stage (42 DAT) to approximately 16.3% at the red ripening stage (84 DAT) (Fig. 3), potentially as a result of the significant decline of cyantraniliprole residues in tomato fruits during the same period (Fig. 1D). In general, our data suggested that ring closure (IN-J9Z38), followed by *N*-dealkylation (IN-MLA84) and glycosylation (TP619) were the predominant transformation pathways of cyantraniliprole across different growth stages of the tomato plants treated through soil drench.

Additionally, there existed many minor metabolites which were detected mostly in the foliage and flowers of the treated tomato plants (Fig. 2). IN-J9Z38 and IN-MLA84 were proposed as the precursors of three dehalogenated metabolites, including IN-RNU71, TP363, and TP423. Their occurrence was observed in both leaves (M/P ratios < 5%) and flowers (trace levels). Reductive dehalogenation is a common transformation pathway of halogenated xenobiotics following plant uptake, as have previously been reported for polychlorinated biphenyls, polybrominated diphenyl ethers, 1,2,5,5,6,9,10-heptachlorodecane, and triclocarban in maize^38^, pumpkin^39,40^, and jalapeno pepper^41^ plants, respectively.

Although hydroxylation of the parent xenobiotics, forming the metabolites that are more suitable for phase-II conjugation reactions, has been frequently observed, this transformation pathway might occur to a negligible extent in tomato plants upon soil application of cyantraniliprole. In this study, IN-MYX98 was the only hydroxylated cyantraniliprole detected in the tomato leaves and flowers, with the M/P ratios of < 0.5%. Similarly, hydroxylation at the cyano group to amide (IN-JCZ38) and carboxylic acid (IN-JSE76) metabolites also exhibited the highest M/P ratios of approximately 2.1% and 0.6% in mature leaves, respectively. Only trace levels of IN-JCZ38 were present in the flowers, while none of these hydroxylated metabolites were detectable in fruits at any developmental stages. In addition to TP619, other minor glycosylated conjugates were also observed, including TP633, TP651a, TP651b, and TP654. As shown in Fig. 3, these metabolites were largely present in mature leaves, with M/P ratios ranging 0.1–3.4%. Only TP651a was detected in flowers with M/P ratios of 0.3–0.8%.

In addition to the above-mentioned phase-I and phase-II metabolites, we also observed the presence of some breakdown products, such as IN-DBC80, IN-M2G98, and TP315 (Fig. 2). IN-DBC80 was detected at higher abundances in young leaves compared to mature leaves in all sampling intervals (Fig. 3). Its highest M/P ratios in young leaves were approximately 2.2% at 49 DAT, while the abundances in mature leaves were negligible. Interestingly, IN-DBC80 also exhibited similar M/P ratios in flowers, reaching approximately 2.5% at 49 DAT, and subsequently was found as the most abundant metabolite in fruits, with the M/P ratios ranging from 6.6 to 31.7% during 42–77 DAT; however, the M/P ratio of IN-DBC80 was abruptly decreased to approximately 9.5% at the red ripening stage (84 DAT). Another frequently detected breakdown metabolite was TP315, which was present in leaves and flowers (M/P ratios of < 2%), but undetectable in fruits at all sampling intervals.

Collectively, the results obtained from this study indicated that cyantraniliprole is relatively recalcitrant to plant metabolism. Over the course of the experiment, cyantraniliprole was the major residue in different tissues up to fruit maturity. The biotransformation processes primarily occurred in the above-ground biomass, especially in the mature leaves. Cyantraniliprole → IN-J9Z38 → IN-MLA84 → TP619 were the predominant transformation pathways of cyantraniliprole in tomato plants, as evident by their ubiquitous presence in leaves, flowers, and fruits at different times after treatment. Additionally, biotransformation of cyantraniliprole in tomato plants was also associated with several metabolites, including phase-I, phase-II, and other breakdown products, in low abundance. To our best knowledge, this study captures for the first time the metabolic fate of a diamide insecticide over the lifespan of a crop plant.

In this study, the highest cyantraniliprole concentrations were approximately 5.3 ng/g in green fruits and 1.4 ng/g in ripening fruits, which were substantially lower than the Maximum Residue Limit set by the U.S. EPA for fruiting vegetables (2000 ng/g)^32^. Recently, there have been growing concerns about human health risks associated with the presence of xenobiotic metabolites in plants^42^. IN-J9Z38 has frequently been observed as a major metabolite via abiotic degradation and plant metabolism of cyantraniliprole, and is required to be reported as the residue of concern for risk assessment in processed commodities^32,36,43^. Our data indicated that metabolism of cyantraniliprole in plants via soil drench occurred slowly, in which the M/P ratios of IN-J9Z38 in mature leaves were approximately 5.3% at 28 DAT. On the other hand, IN-J9Z38 was undetectable in fruits. It has been known that glycosylated metabolites are likely to be hydrolyzed, or further transformed by the gut microbes during digestion^44^. However, the metabolite TP619 was only present in the fruits at trace levels, suggesting that it is of a minor concern. Accordingly, in tomatoes, cyantraniliprole residue should be the focus for risk assessment, since none of the metabolites seems to accumulate in fruits.

## Materials and methods

Standard of cyantraniliprole (purity 98.2%) was purchased from Chemservice (West Chester, PA). The isotope-labeled *d*_3_-cyantraniliprole (purity > 95%) was purchased from Clearsynth (Mississauga, Ontario, Canada). MS-grade water, acetonitrile, and formic acid were purchased from Fisher Scientific (Waltham, MA). Analytical grade anhydrous sodium acetate (CH_3_COONa) and anhydrous magnesium sulfate (MgSO_4_) were purchased from Sigma-Aldrich (St. Louis, MO). C18 endcapped bulk sorbent (Part No. 5982-5752) was obtained from Agilent Technologies (Santa Clara, CA).

### Plant growth and insecticide application

The experiments were conducted in a greenhouse at Clemson University (Clemson, SC) from October 2019 to February 2020. The greenhouse was under natural light and maintained at average min–max temperature of 24–27 °C, and relative humidity of 60–70% during the treatment period. Tomato seeds (cv. “Black Sea Man”) were obtained from Seed ‘n Such (Graniteville, SC) and were sown three seeds in each plastic pot (28 cm diameter, 24 cm depth) filled with a standard nursery mix made of 55% peat moss, 30% bark, and 15% perlite (Metro-Mix 830; Sun Gro Horticulture, Agawam, MA). Germination was observed 5–7 days after sowing. Subsequently, seedlings at 2–3 leaf stage were thinned to one plant per pot. Plants were hand-watered as needed and fertilized with Miracle-Gro^®^ All Purpose fertilizer (24–8–6) every 1–2 weeks after application of cyantraniliprole until the conclusion of the experiments.

Tomato plants were treated with cyantraniliprole once via soil drench approximately four weeks after germination, using low application rate recommended for greenhouse-grown plants. Mainspring^®^ GNL (18.66% cyantraniliprole; Syngenta Crop Protection LLC, Greensboro, NC) was diluted in water at the application rate of 0.63 mL/L (8 fl. oz. formulated product per 100 gal.). Each pot in the treated group received 470 mL of the diluted solution (approximately 59 mg of cyantraniliprole per pot). The control group received the same amount of water. Each group consisted of 40 plants, which were arranged in a completely randomized design on a greenhouse bench.

### Plant tissue harvest

**Figure 4:**
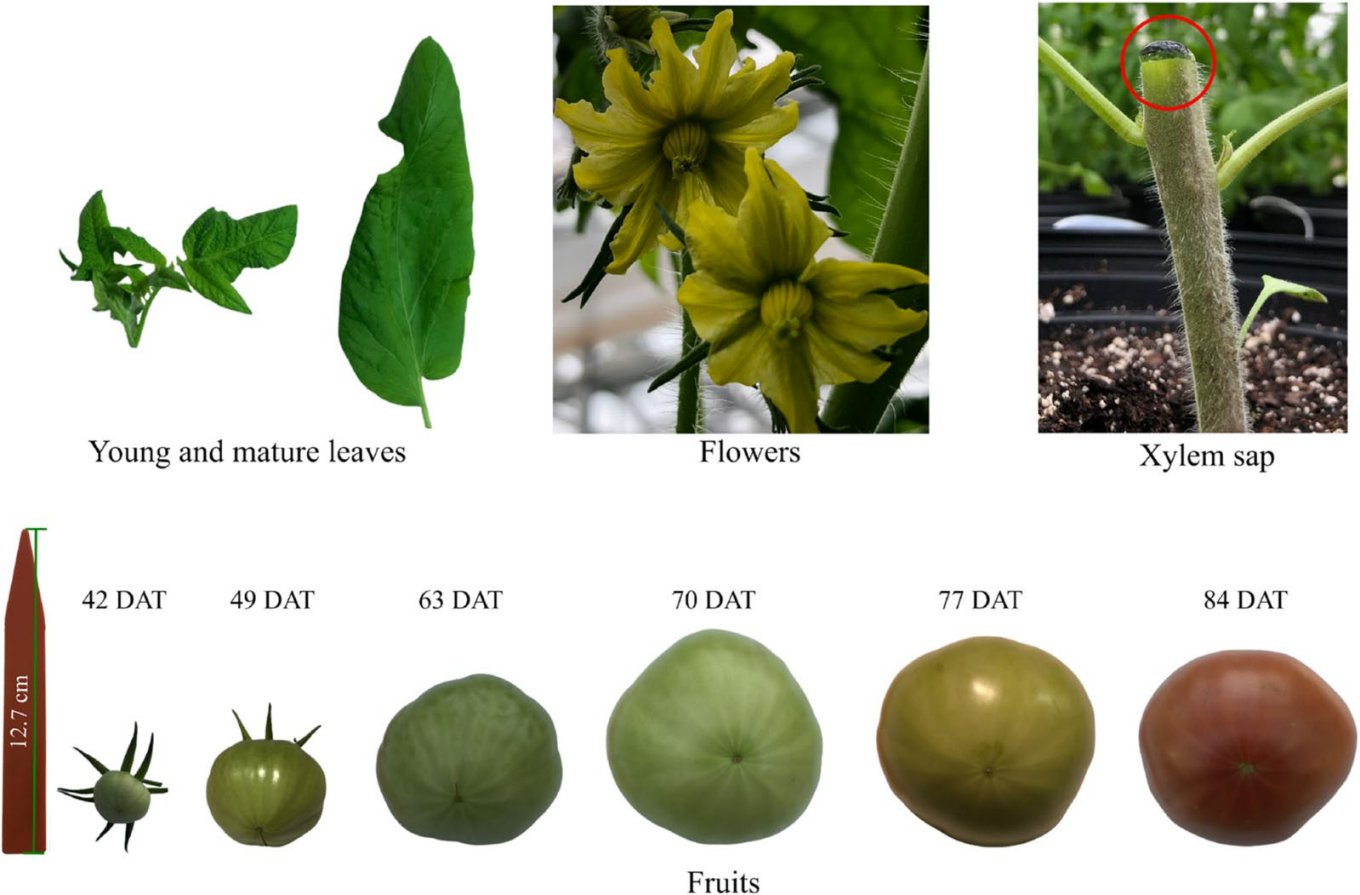
Sampling scheme showing tomato young and mature leaves, flowers, xylem sap, and fruits at different developmental stages. *DAT* days after treatment.

Xylem sap, leaves, flowers, and fruits were collected at 7- to 14-day intervals from 7 to 84 DAT (Fig. 4, Table S1). At each interval (except 63 and 70 DAT), 5 plants from each treatment were randomly selected and destructively sampled for both mature and young leaves. Mature, fully expanded leaves at different sides of the middle section of the plants were sampled, whereas young leaves (apical leaves) were collected from top of the plants. Three to four leaves were harvested from each plant and combined to form two composite samples per plant—one representing young leaves and the other representing mature leaves. Flowers were only sampled at full-open (anthesis) stage from 14 to 49 DAT. Fruits were harvested from immature green (42 DAT) to red ripening (84 DAT), in which 2–3 fruits of similar size were collected from each plant at each sampling interval. The samples were kept on dry ice before transport to the laboratory and stored at − 80 °C until analysis.

Collection of xylem sap was performed as previously described by Lowe-Power et al.^45^. Plants were well-watered each evening before morning sampling to build up root pressure. One to three hours after sunrise, the plants were detopped at approximately 7 cm above the soil surface using a sharp razor to yield a flat stump surface, on top of which xylem sap accumulated as a droplet. To minimize contamination, the sap droplet accumulated in the first 2 min of detopping was discarded, and the stump was washed with deionized water and gently blotted dry with Kimwipes tissues. For the next 20 min, the sap was frequently pipetted into a 2.0-mL centrifuge tube kept on ice. The tubes were then stored at − 80 °C until analysis.

### Sample preparation

Xylem sap was analyzed without further preparation. Leaves and flowers (approximately 0.5 g per sample) were weighed into 7-mL tubes. Prior to extraction, internal standard *d*_3_-cyantraniliprole was spiked into each sample at 120 ng/g fresh weight (fw). Sample homogenization was facilitated with the addition of 5 ceramic beads, 1.5 mL water, and 3.0 mL acetonitrile. The samples were then thoroughly homogenized at 6000 rpm for 4 repetitions of 30-s cycles using a homogenizer (Precellys^®^ Evolution, Bertin Instruments). The samples were centrifuged at 3000 rpm for 5 min, and the supernatant was transferred into new tubes containing 800 ± 5 mg MgSO_4_ anhydrous and 200 ± 5 mg CH_3_COONa anhydrous. The samples were vortexed at 2500 rpm for 10 min and centrifuged at 3000 rpm for 5 min to obtain transparent acetonitrile phase. For matrix removal, aliquots of 1.0 mL of the acetonitrile supernatant were transferred into clean 2.0-mL tubes containing 200 ± 5 mg C18 sorbent and 150 ± 5 mg MgSO_4_ anhydrous. The samples were shaken on a rotary shaker at 50 rpm for 5 min and centrifuged at 14,000 rpm for 10 min. Finally, the supernatant was transferred into 2.0-mL glass vials. For flowers, an additional concentration step (5 times) was performed by nitrogen blow-down and reconstitution to accurately verify the presence of low-abundant metabolites in the samples.

Fruits were homogenized in a blender, and aliquots of 5.0 g pulp were weighed into 50-mL tubes. Prior to extraction, *d*_3_-cyantraniliprole was spiked into each sample at a concentration of 4 ng/g fw. Acetonitrile (10 mL) was then added into each tube and the samples were vortexed at 2500 rpm for 10 min. Next, 4.0 ± 0.1 g of MgSO_4_ anhydrous and 1.0 ± 0.1 g of CH_3_COONa anhydrous were added, and the mixture was vortexed again for 10 min and centrifuged at 3000 rpm for 5 min. Aliquots of 1.5 mL of the supernatant were cleaned up with C18 sorbent and MgSO_4_ anhydrous as described above. Finally, 1.0 mL clean supernatant were transferred into 2.0-mL glass vials, dried under nitrogen gas, and reconstituted in 0.1 mL acetonitrile.

### Cyantraniliprole quantification and metabolites screening

The extracts were analyzed on an Ultimate 3000 HPLC (Thermo Scientific, Waltham, MA) coupled with an Orbitrap Fusion Tribrid mass spectrometer (Thermo Scientific). Chromatographic separation was achieved using Acquity HSS-T3 column (150 × 2.1 mm, 1.8 μm; Waters Corp., Milford, MA) maintained at 32 °C. The mobile phases consisted of (A) water and (B) acetonitrile, both containing 0.1% formic acid. The gradient program was: 0–1 min, 20% B; 9 min, 95% B; 9–12 min, 95% B; 12.01 min, 20% B; and re-equilibration at 20% B for 4 min. The flow rate was maintained at 0.2 mL/min and the injection volume was 2 µL. The autosampler temperature was maintained at 8 °C. The samples were introduced into mass spectrometer using a heated electrospray ionization (H-ESI) source operated in positive ionization mode. The H-ESI interface parameters were as follows: spray voltage, 3.5 kV; vaporizer, 300 °C; ion transfer tube, 300 °C; sheath gas, 55 arb; aux gas, 10 arb; and sweep gas, 1 arb. XCalibur™ 4.0 software (Thermo Scientific) was used for data acquisition.

Data were initially acquired using a full MS^1^ scan on the Orbitrap within a mass range of 150–1200 m*/z*, with scan resolution set to 120,000 (FWHM), AGC of 400,000, and a maximum injection time of 50 ms. The acquired data were used for primary screening of the metabolite candidates. Fragmentation of the metabolite candidates was subsequently performed using data-dependent MS^2^ collision-induced dissociation (CID) at 40% collision energy. The MS^1^ master scan was operated with a scan range of 120–1000 m*/z,* with an Orbitrap resolution of 60,000 (FWHM), AGC of 200,000 and maximum injection time of 50 ms. The data-dependent MS^2^ scan was performed for the *m/z* values listed in the targeted mass inclusion filter containing all metabolite candidates with a mass tolerance of 25 ppm. The scan resolution for MS^2^ was set to 15,000 (FWHM), AGC of 100,000, and maximum injection time of 54 ms.

All samples were immediately analyzed after extraction in one single batch, with QC sample containing cyantraniliprole at 12.5 ng/mL injected after every 15 samples. The relative standard deviations of all QCs were from 1.3 to 5.9%. Quantification of cyantraniliprole residue in plant tissues was performed using data acquired from Orbitrap MS^1^ scan in selected ion monitoring mode. Cyantraniliprole was identified by retention time (RT ± 0.1 min), accurate molecular ion at *m/z* 473.0123, and reference isotope ions at *m/z* 475.0101 and 477.0074 (mass errors < 5 ppm). Stock solutions of cyantraniliprole and *d*_3_-cyantraniliprole (1 µg/mL) were prepared in acetonitrile and stored at − 20 °C. Working standards (0.8–800 ng/mL) were freshly prepared prior to instrument analysis.

Recoveries of cyantraniliprole and potential plant matrix interference on quantification were determined by the ratio of internal standard *d*_3_-cyantraniliprole concentration in the final extract to the nominal spiked concentration in each sample (e.i., 120 ng/g for leaves and flowers, and 4 ng/g for fruits, respectively). Our preliminary trials indicated that the conventional QuEChERS matrix removal procedure resulted in significant loss of some cyantraniliprole metabolites (e.g., glycosylated conjugates). Therefore, in this study, matrix was partly removed using only C18 sorbent to essentially preserve the metabolite pool. As a result, plant matrix effects on quantitative analysis were still observed to different extent depending on the type and age of the tissues. Consequently, the concentration of cyantraniliprole was corrected to the respective recovery rate of *d*_3_-cyantraniliprole in each sample by multiplying the initial result obtained after analysis with a factor [100%/recovery %]^46^. In this study, the mean recoveries of *d*_3_-cyantraniliprole for young leaves, mature leaves, flowers, green fruits, and red fruits were 92.6 ± 8.3, 81.0 ± 9.9, 105.7 ± 8.5, 74.1 ± 17.1, and 63.2 ± 14.5%, respectively.

Limit of detection (LOD) of cyantraniliprole in each plant matrix was determined using spiked samples approach^47^. The untreated sample matrices were spiked with cyantraniliprole at a nominal concentration of 2 ng/mL which was approximately five times the initial instrument LOD. The method LOD was then determined as follow:

$${\text{LOD}}\left( {\text{ng/mL}} \right) = {\text{t}}_{\upalpha } \times S_{s}$$

where t_α_ is the Student’s *t*-value appropriate for a single-tailed 99th percentile *t* statistic and a standard deviation estimate with n − 1 degrees of freedom; for seven injections, t_α_ = 3.143.* S*_*s*_ is the sample standard deviation of the replicate spiked sample analyses. Limit of quantification (LOQ) = 3.3 × LOD.

The LOD and LOQ values of cyantraniliprole in different plant matrices and fresh biomass are provided in Table S2. On a fresh weight basis, the LOQ in young leaves, mature leaves, flowers, green fruits, and red fruits were 5.6, 6.2, 7.7, 0.3, and 0.4 ng/g, respectively.

Concentrations of cyantraniliprole in xylem sap were quantified on a Shimadzu Prominence UFLC coupled with a Shimadzu 8040-triple quadrupole mass spectrometer, applying ESI in positive mode. Chromatographic separation was performed at room temperature on a Kinetex XB-C18 column (150 × 3 mm, 2.6 μm, Phenomenex). Water and acetonitrile, both containing 0.1% formic acid, were used as the mobile phase A and B, respectively. The gradient program was: 0–2 min: 15% B; 8 min, 95% B; 8–11 min: 95% B; 11.01 min: 15% B; and re-equilibration at 15% B for 5 min. The flow rate was maintained at 0.4 mL/min and the injection volume was 2 µL. The interface voltage was set to 4.5 kV and nitrogen was used as nebulizing gas (3 L/min). The desolvation line and heat block temperatures were set to 250 and 400 °C, respectively. The MRM transition *m/z* 474.9 → 285.9 (CE = 15 V) was used for quantification, while the transitions *m/z* 474.9 → 176.9 (CE = 49 V) and *m/z* 474.9 → 111.9 (CE = 55 V) were used for confirmation. The LOD and LOQ values of cyantraniliprole in xylem sap were 0.3 and 1.1 ng/mL, respectively.

### Data processing for metabolite identification

Screening of cyantraniliprole metabolites was performed as previously described^18^. Briefly, MS^1^ scan data were processed by Compound Discoverer 3.1 software (Thermo Scientific), using a custom designed workflow as shown in Fig. S1. The *m/z* signals present in the nontreated samples were eliminated from being screened as cyantraniliprole metabolites. The metabolite candidates were further processed via a Cl/Br isotope filter, assuming the metabolites contained at least one Cl and/or one Br atom from the parent cyantraniliprole. Subsequently, Mass Frontier 8.0 software (Thermo Scientific) was used to generate the possible chemical formulas for the metabolite candidates identified by Compound Discoverer, with mass-accuracy errors of < 5 ppm for the molecular ion [M + H]^+^. Following the primary identification of the metabolite candidates, the proposed structures were further confirmed by the characteristic fragment ions (mass-accuracy errors < 5 ppm) acquired by data-dependent MS^2^ fragmentation. Annotation of the fragment ions was performed using Mass Frontier 8.0 and CFM-ID 3.0 web server^48^. The identification confidence was ultimately classified following the framework proposed by Schymanski^30^.

A list of cyantraniliprole metabolites was compiled from the literature (Table S3). The metabolites identified by Compound Discoverer were subsequently searched against the compiled list of metabolites using their accurate *m/z* and the corresponding MS^2^ fragment ions. The remaining metabolites were annotated as newly identified transformation products (TPs). Due to the lack of reference standards, relative quantification of the tentatively identified metabolites (intensity cutoff at approximately 10^4^, with signal-to-noise ratio > 10) compared to the parent cyantraniliprole were reported. The chromatograms of cyantraniliprole and metabolites at the levels of quantification are provided in Figs. S23 and S24.

### Statistical analysis

Concentrations of cyantraniliprole in plant tissues were calculated based on fresh weight (fw). Analysis of variance (ANOVA) with Sidak’s or Tukey’s HSD post hoc tests were performed using GraphPad Prism 8.4.3 (GraphPad Software, San Diego, CA) to detect significant differences and separate means among plant tissues and sampling intervals (*α* = 0.05).

### Ethics statement

The tomato seeds used in this study is commercially available in the United States; therefore, this study does not contain any research requiring ethical consent or approval. Insecticide treatments and collection of plant samples complied with relevant institutional, national, and international guidelines and legislation.

## Supplementary Information

Supplementary Information.

## Data Availability

Data supporting the findings of this manuscript are available from the corresponding author upon reasonable request.
