# Supplementary material for: Persistence and metabolism of the diamide insecticide cyantraniliprole in tomato plants
Source: Sci Rep. 2021 Nov 3;11:21570. doi: 10.1038/s41598-021-00970-8 (PMC8566514; doi:10.1038/s41598-021-00970-8)
Supplement: Supplementary file 1 — Supplementary Information. [file 41598_2021_970_MOESM1_ESM.docx]

**Supporting Information**

**Persistence and Metabolism of the Diamide Insecticide Cyantraniliprole in Tomato Plants**

Khang Huynh^1^, Elizabeth Leonard^1^, Juang-Horng Chong^2^, Cristi Palmer^3^, Nishanth Tharayil^1^*

^1^Department of Plant and Environmental Sciences, Clemson University, Clemson, South Carolina 29634, United States

^2^Department of Plant and Environmental Sciences, Pee Dee Research and Education Center, Clemson University, Florence, South Carolina 29506, United States

^3^Rutgers, The State University of New Jersey, IR-4 Project, New Brunswick, New Jersey 08901, United States

*Correspondence author

Phone: (864) 656-4453

Email: ntharay@clemson.edu (Nishanth Tharayil)

34 pages

25 figures

3 tables

| 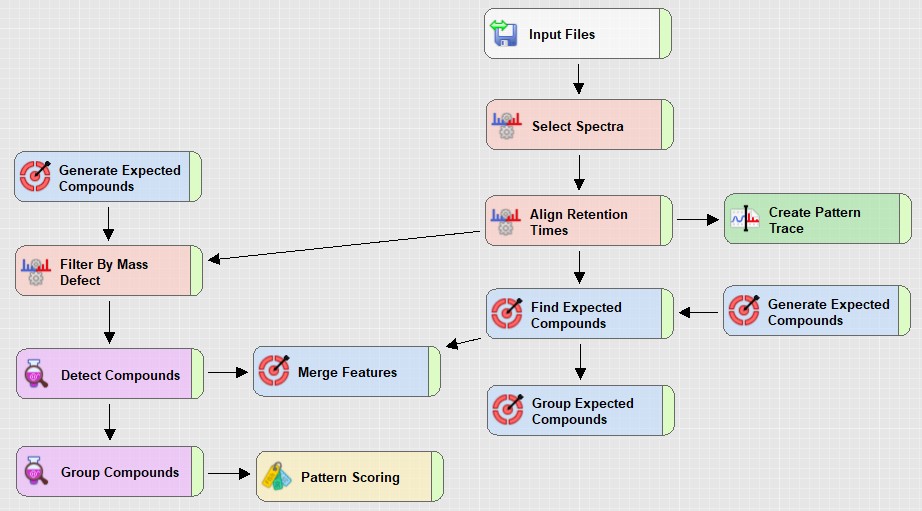 |
| --- |
| **Figure S1.** Workflow tree from Compound Discoverer 3.1 software with data processing nodes and the associated workflow connections. |

| 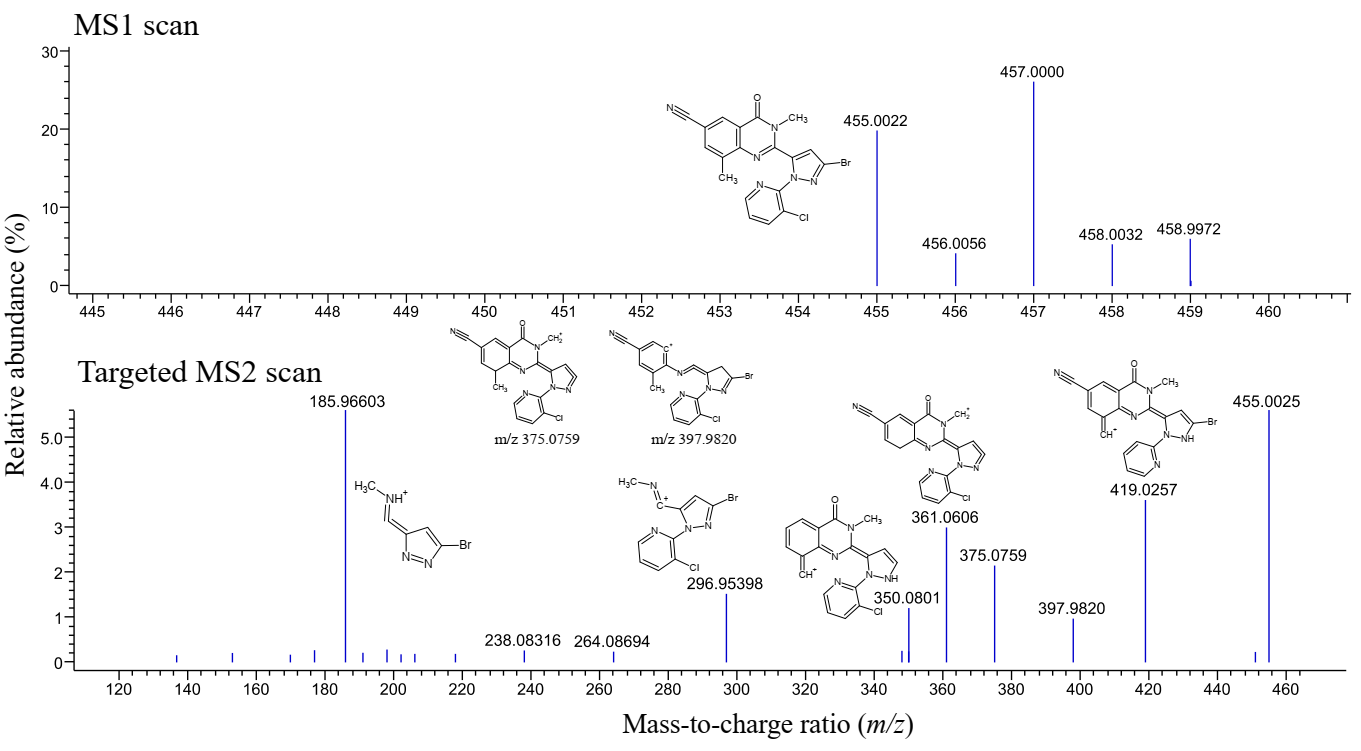 |
| --- |
| **Figure S2.** Mass spectra of IN-J9Z38 on the UPLC-Orbitrap-MS system, acquired using CID mode (mass errors <5 ppm). |

| 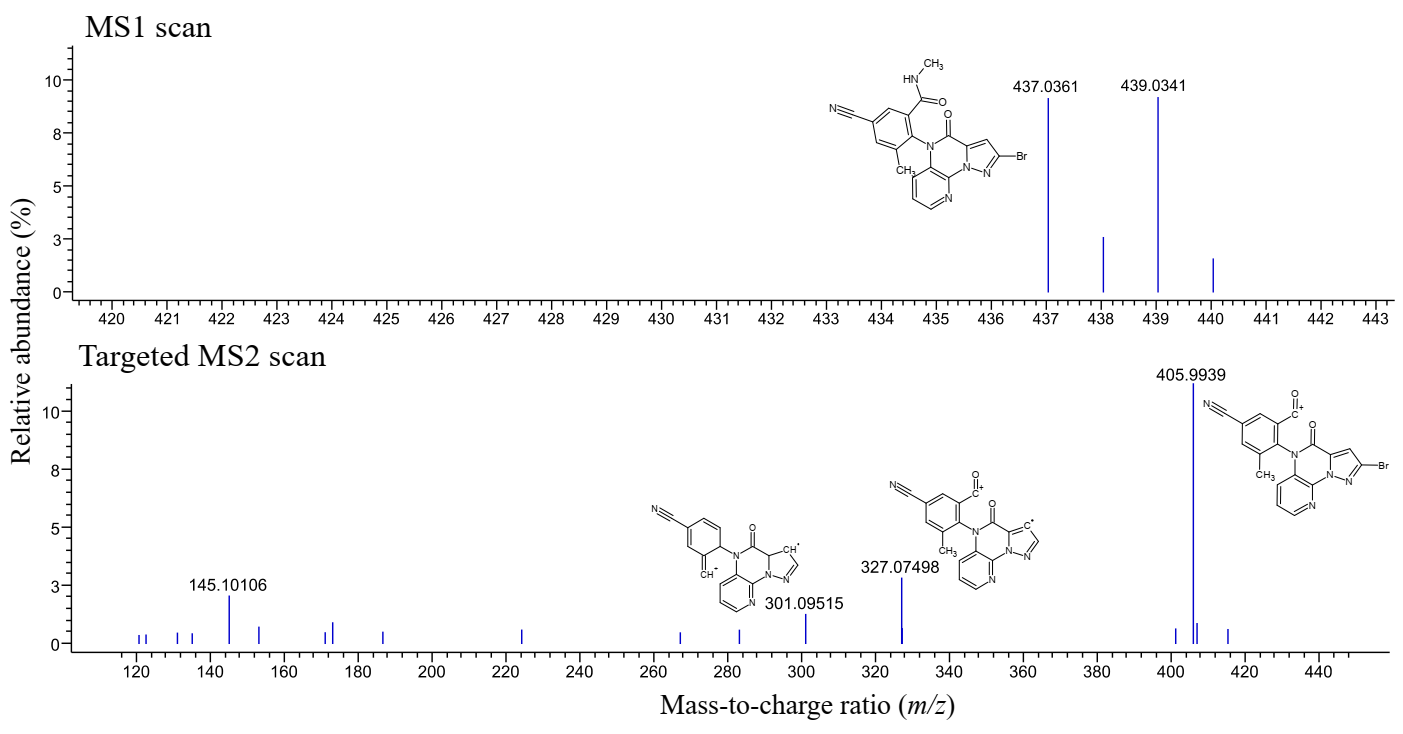 |
| --- |
| **Figure S3.** Mass spectra of IN-RNU71 on the UPLC-Orbitrap-MS system, acquired using CID mode (mass errors <2 ppm). |

| 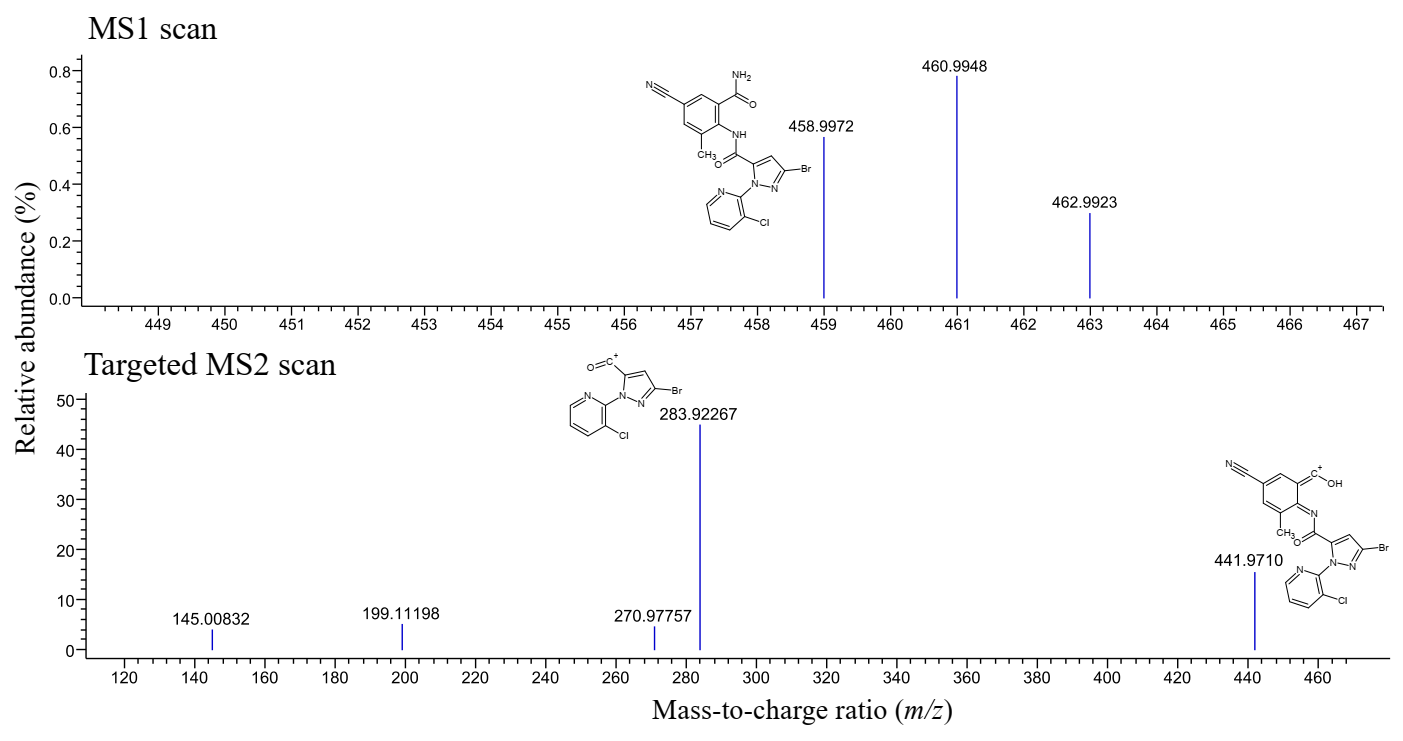 |
| --- |
| **Figure S4.** Mass spectra of IN-HGW87 on the UPLC-Orbitrap-MS system, acquired using CID mode (mass errors <3 ppm). |

| 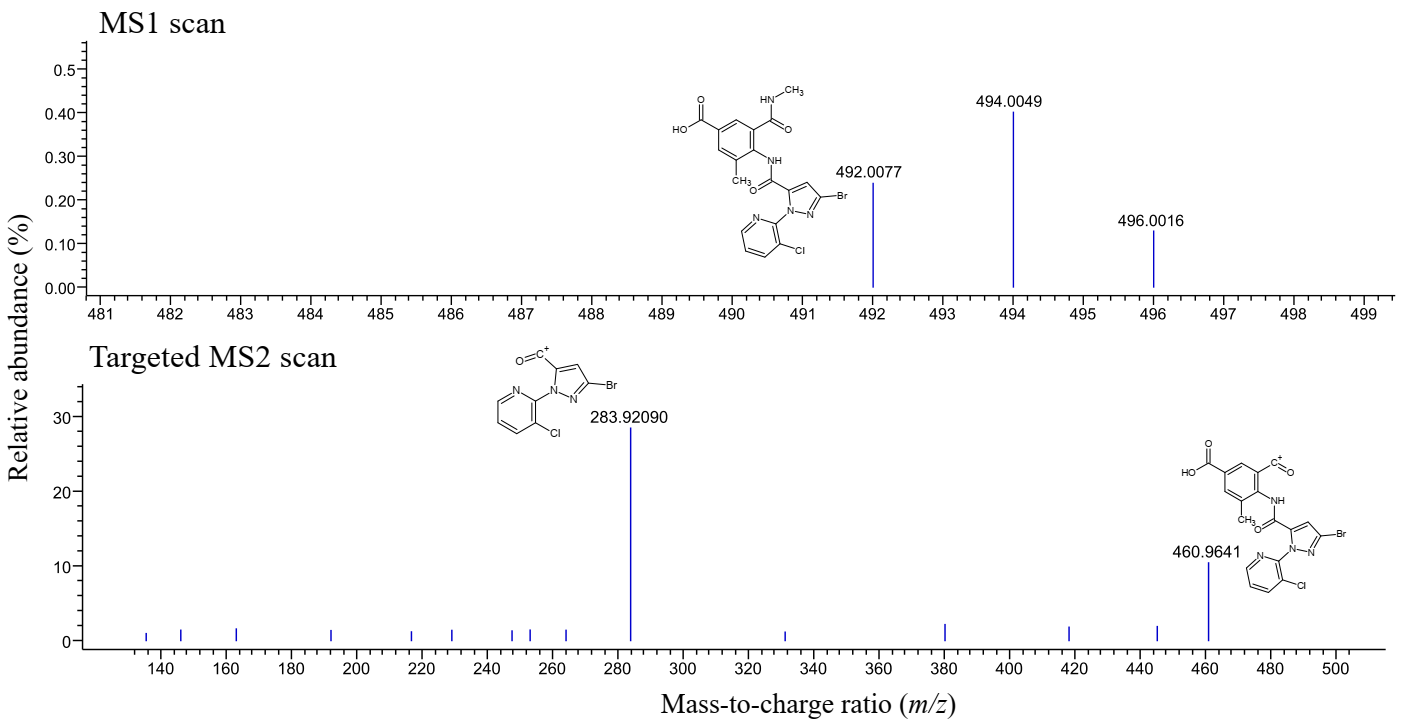 |
| --- |
| **Figure S5.** Mass spectra of IN-JSE76 on the UPLC-Orbitrap-MS system, acquired using CID mode (mass errors <5 ppm). |

| 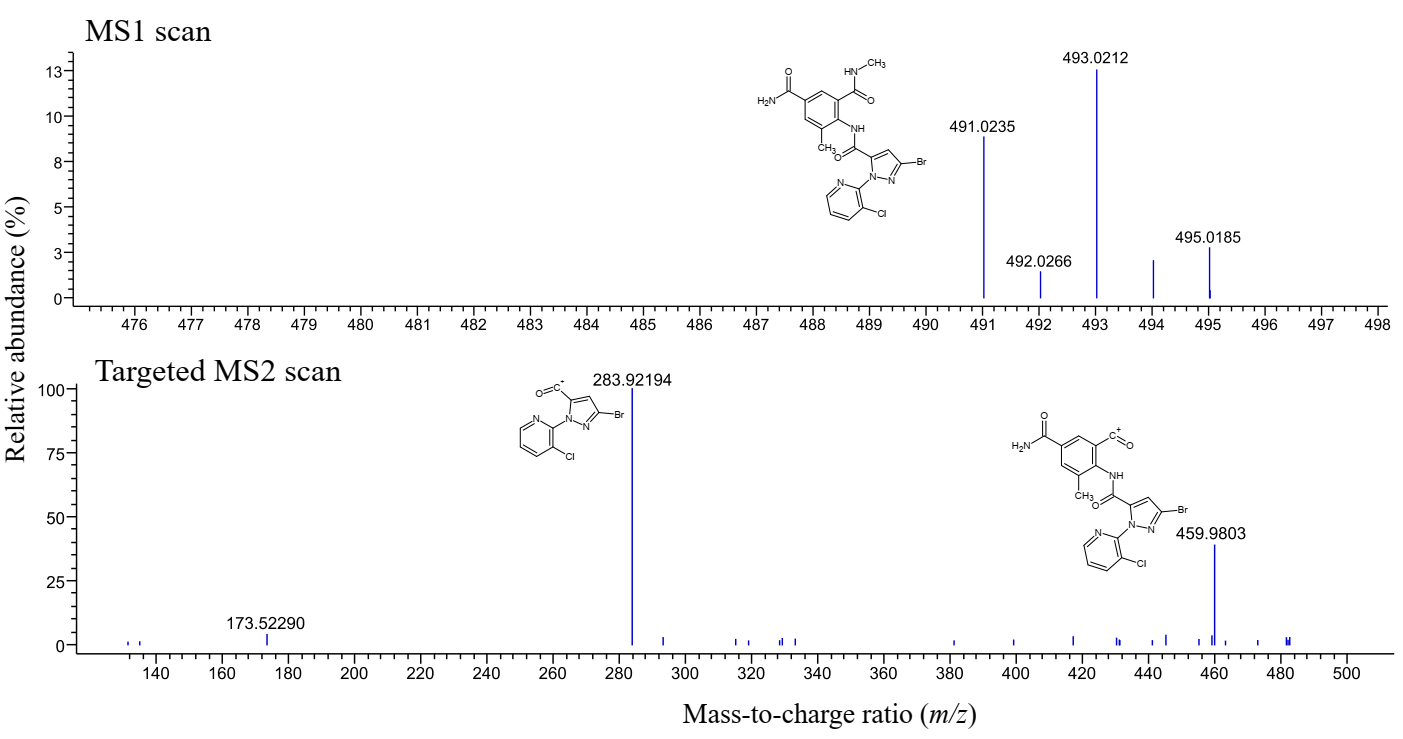 |
| --- |
| **Figure S6.** Mass spectra of IN-JCZ38 on the UPLC-Orbitrap-MS system, acquired using CID mode (mass errors <2 ppm). |

| 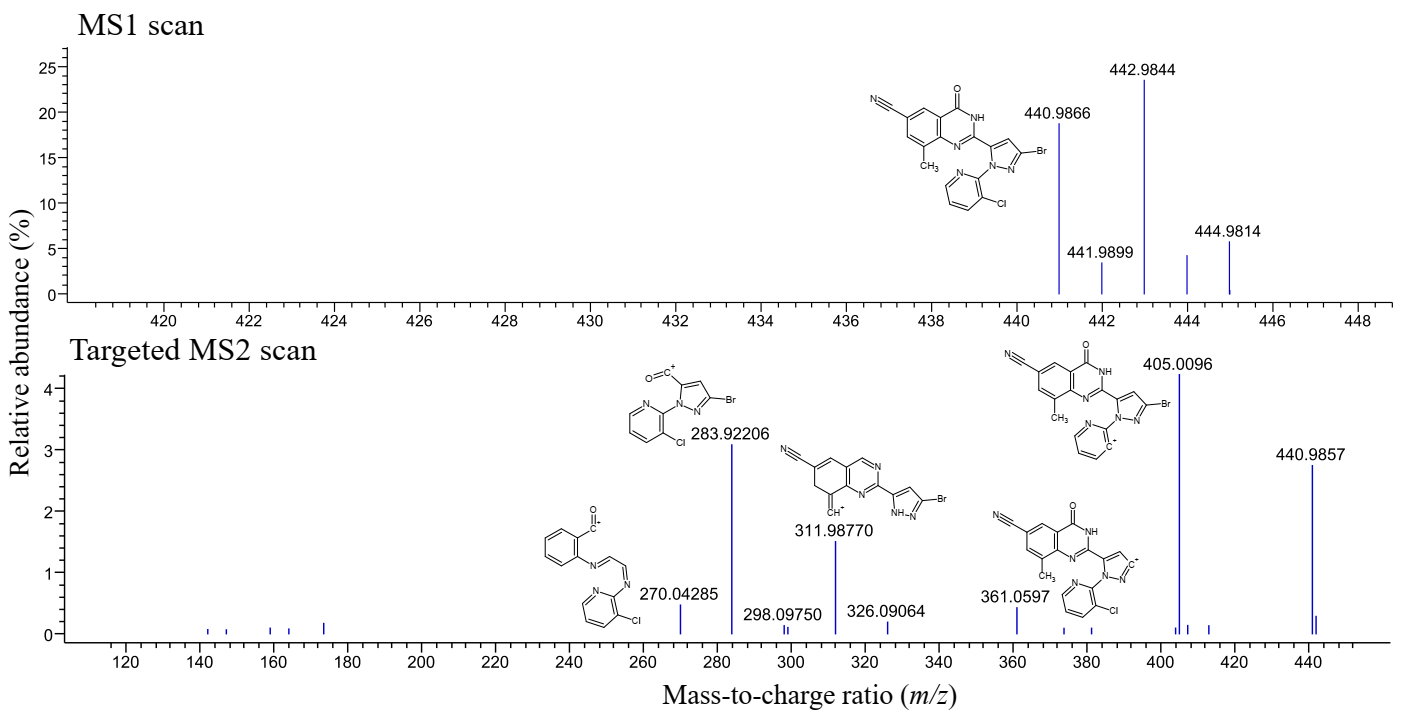 |
| --- |
| **Figure S7.** Mass spectra of IN-MLA84 on the UPLC-Orbitrap-MS system, acquired using CID mode (mass errors <2 ppm). |

| 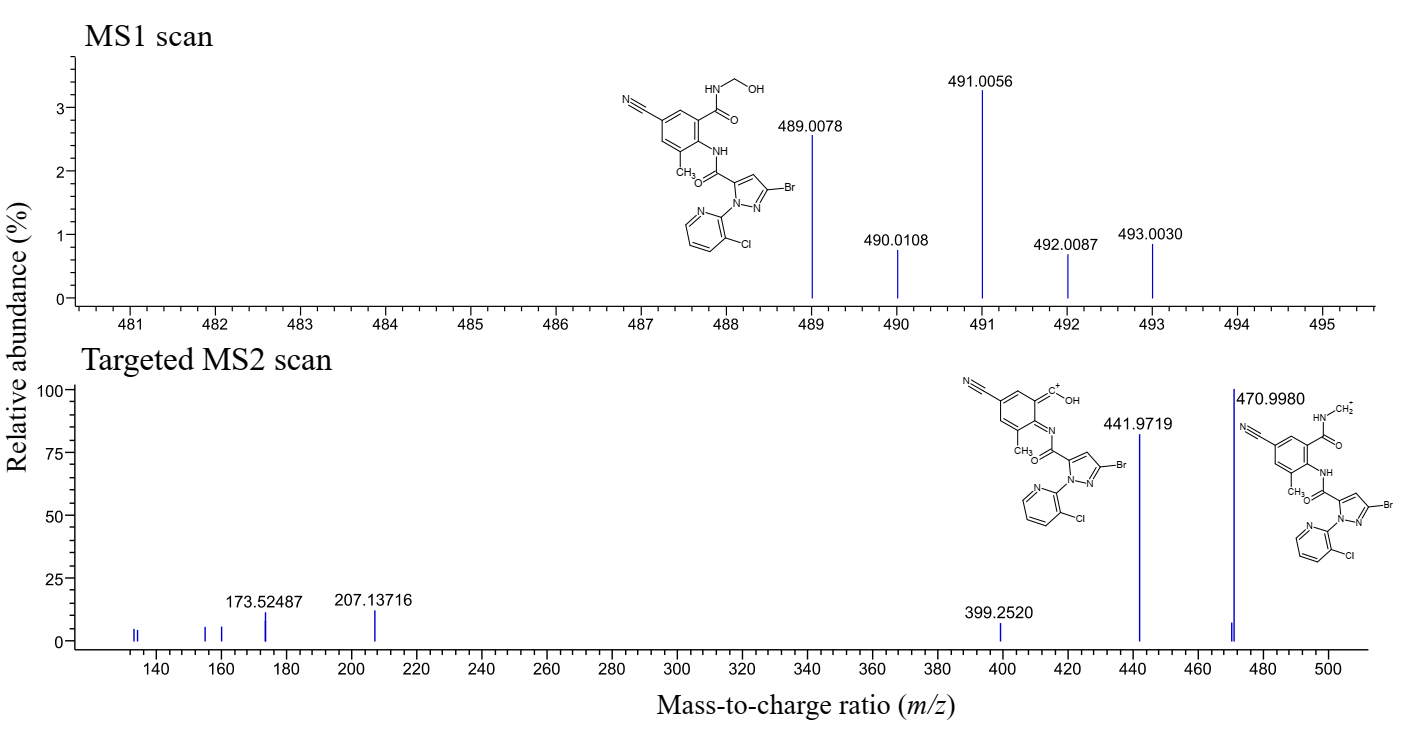 |
| --- |
| **Figure S8.** Mass spectra of IN-MYX98 on the UPLC-Orbitrap-MS system, acquired using CID mode (mass errors <5 ppm). |

| 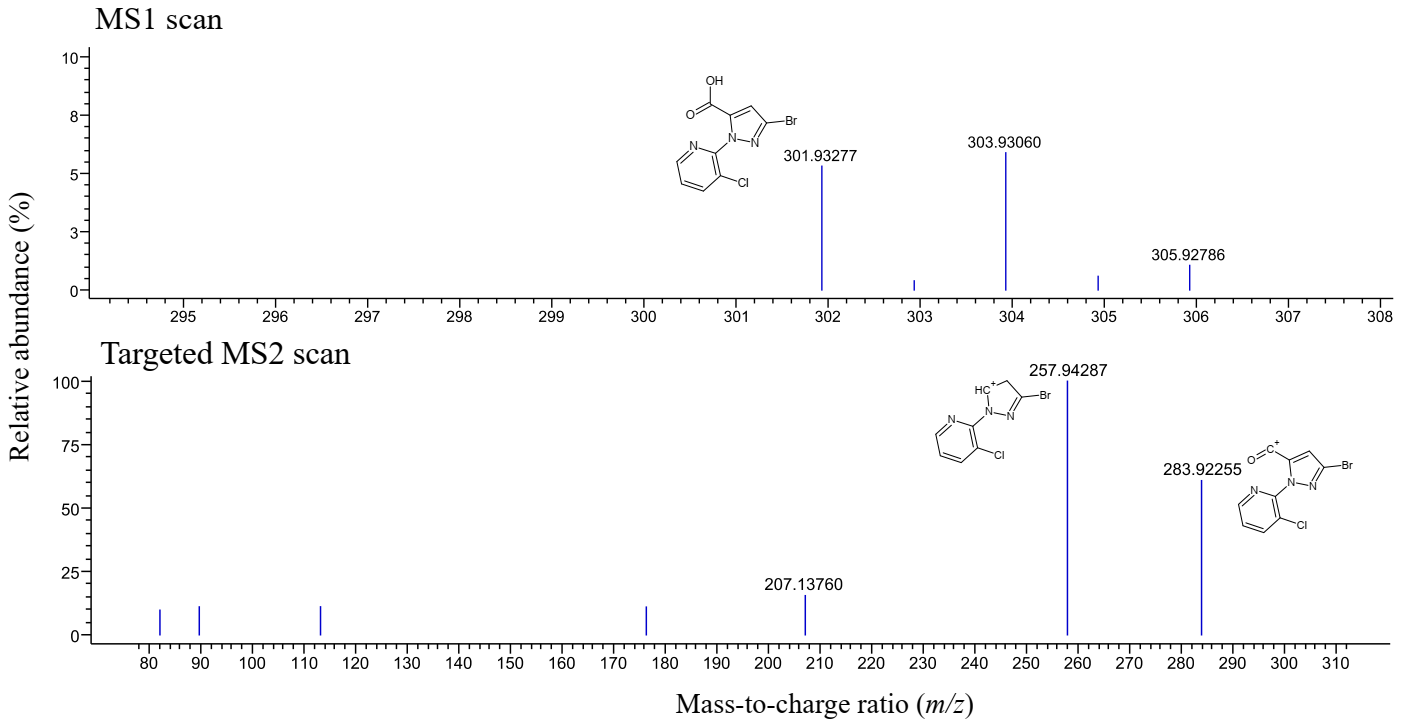 |
| --- |
| **Figure S9.** Mass spectra of IN-DBC80 on the UPLC-Orbitrap-MS system, acquired using CID mode (mass errors <2 ppm). |

| 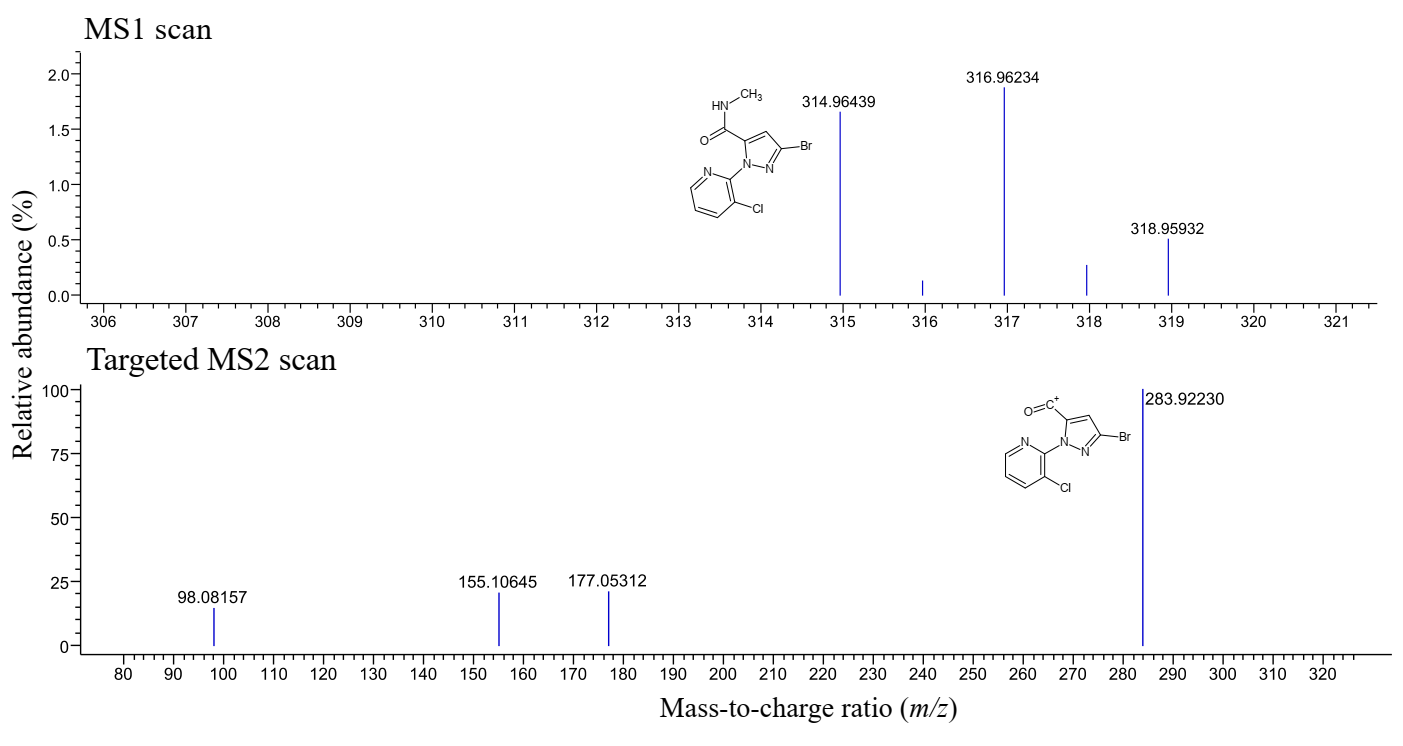 |
| --- |
| **Figure S10.** Mass spectra of TP315 on the UPLC-Orbitrap-MS system, acquired using CID mode (mass errors <1 ppm). |

| 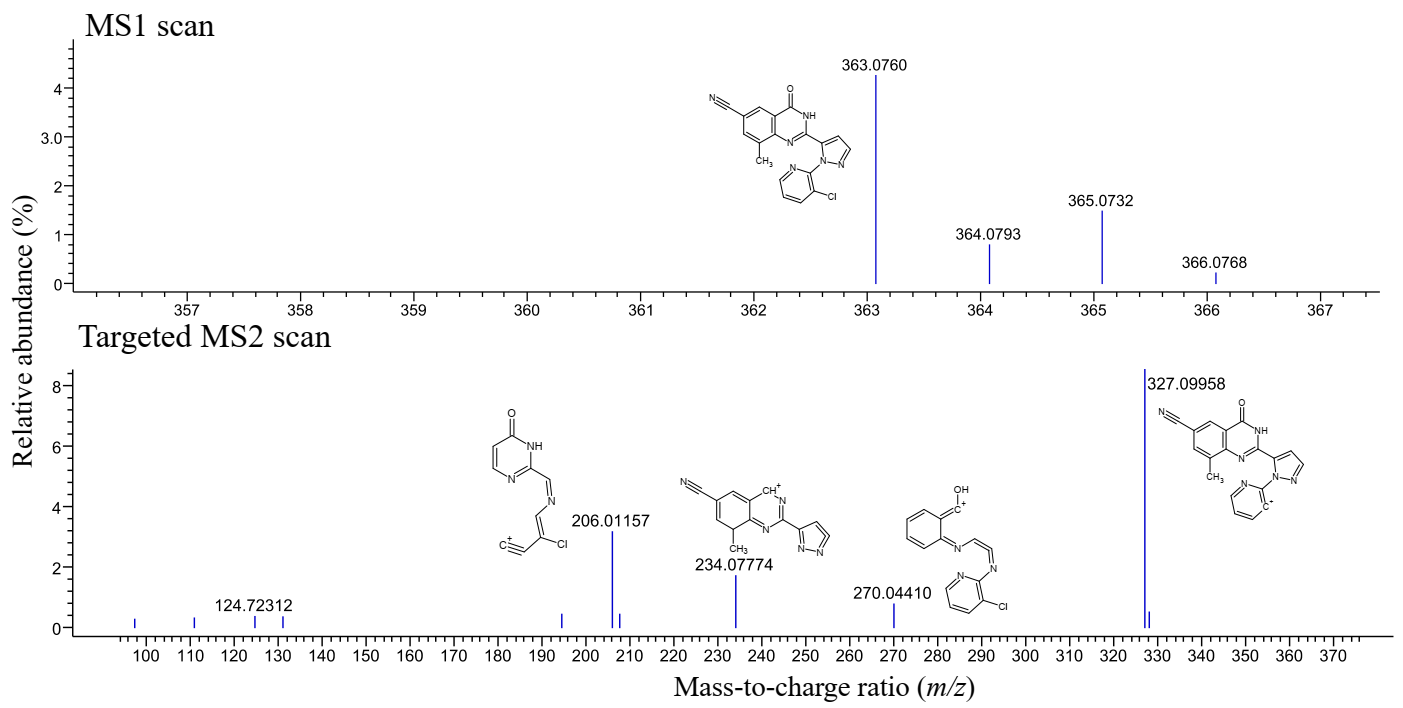 |
| --- |
| **Figure S11.** Mass spectra of TP363 on the UPLC-Orbitrap-MS system, acquired using CID mode (mass errors <5 ppm). |

| 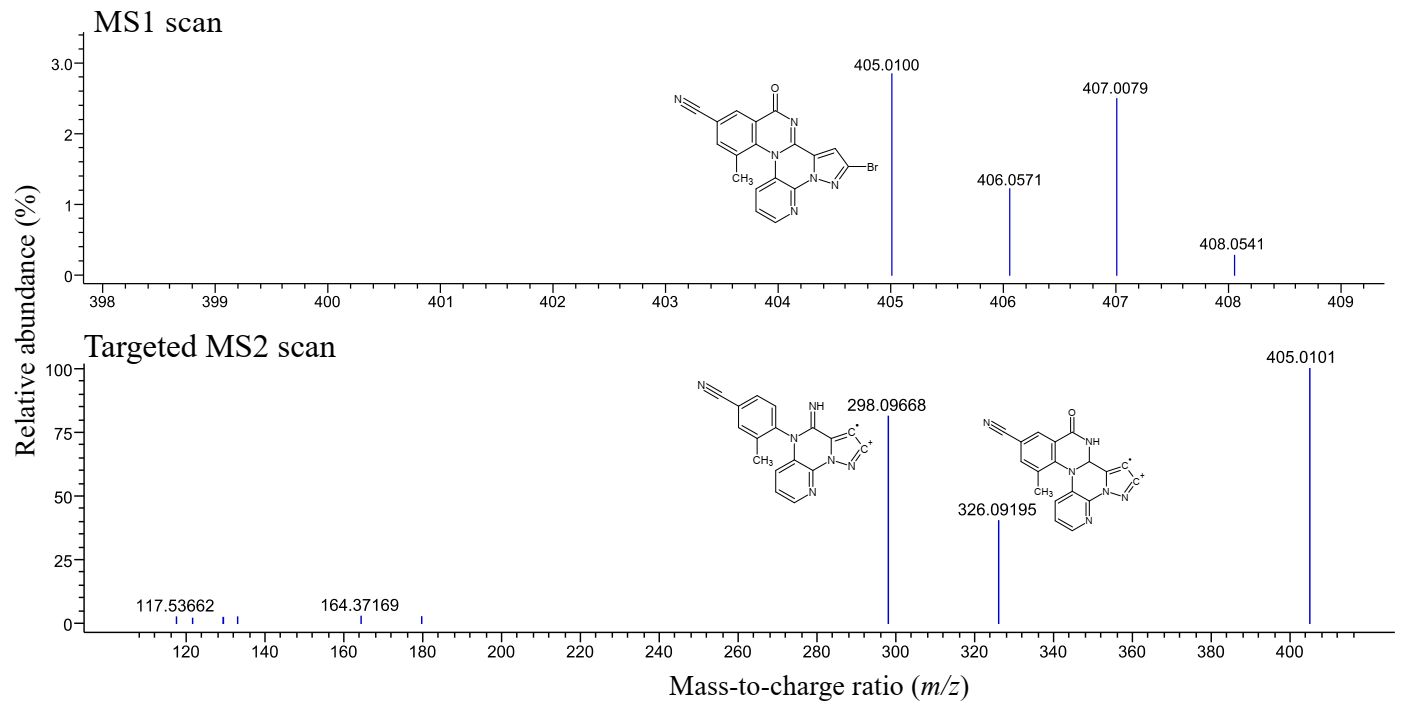 |
| --- |
| **Figure S12.** Mass spectra of TP405 on the UPLC-Orbitrap-MS system, acquired using CID mode (mass errors <3 ppm). |

| 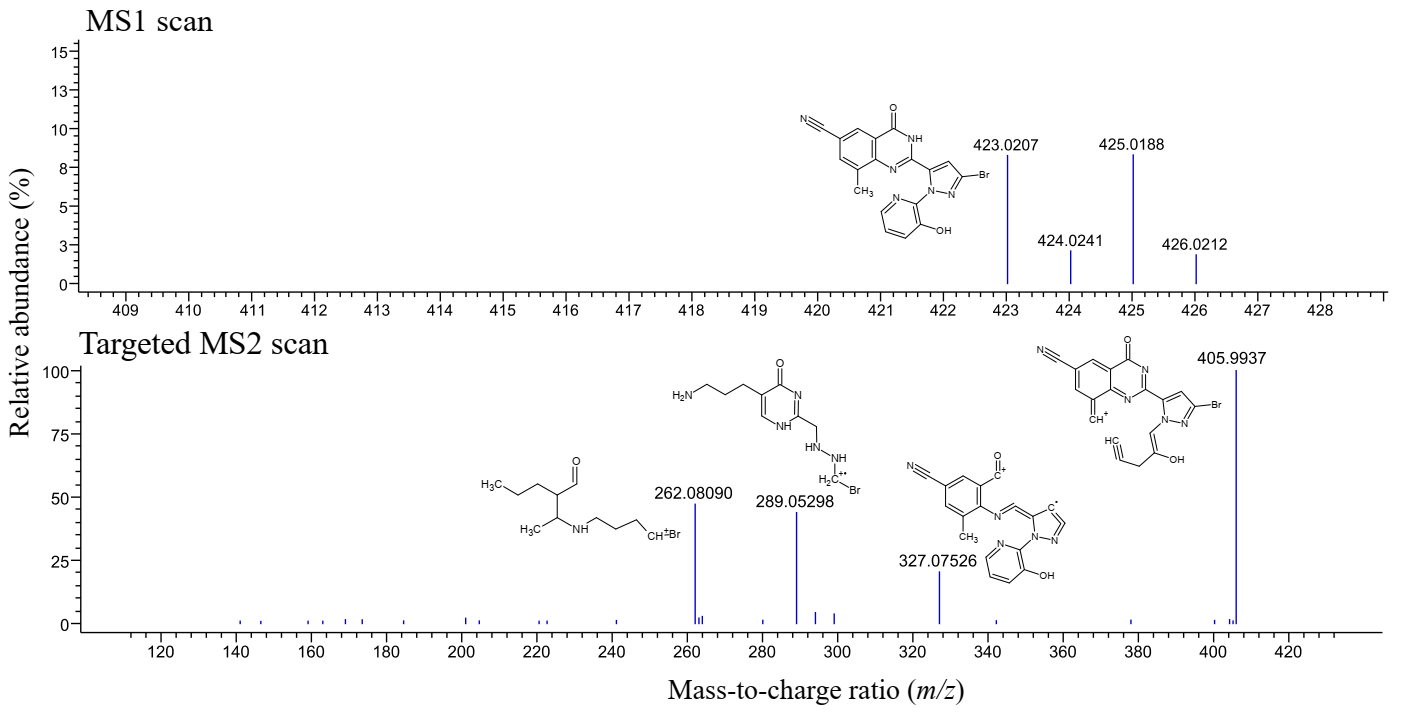 |
| --- |
| **Figure S13.** Mass spectra of TP423 on the UPLC-Orbitrap-MS system, acquired using CID mode (mass errors <4 ppm). |

| 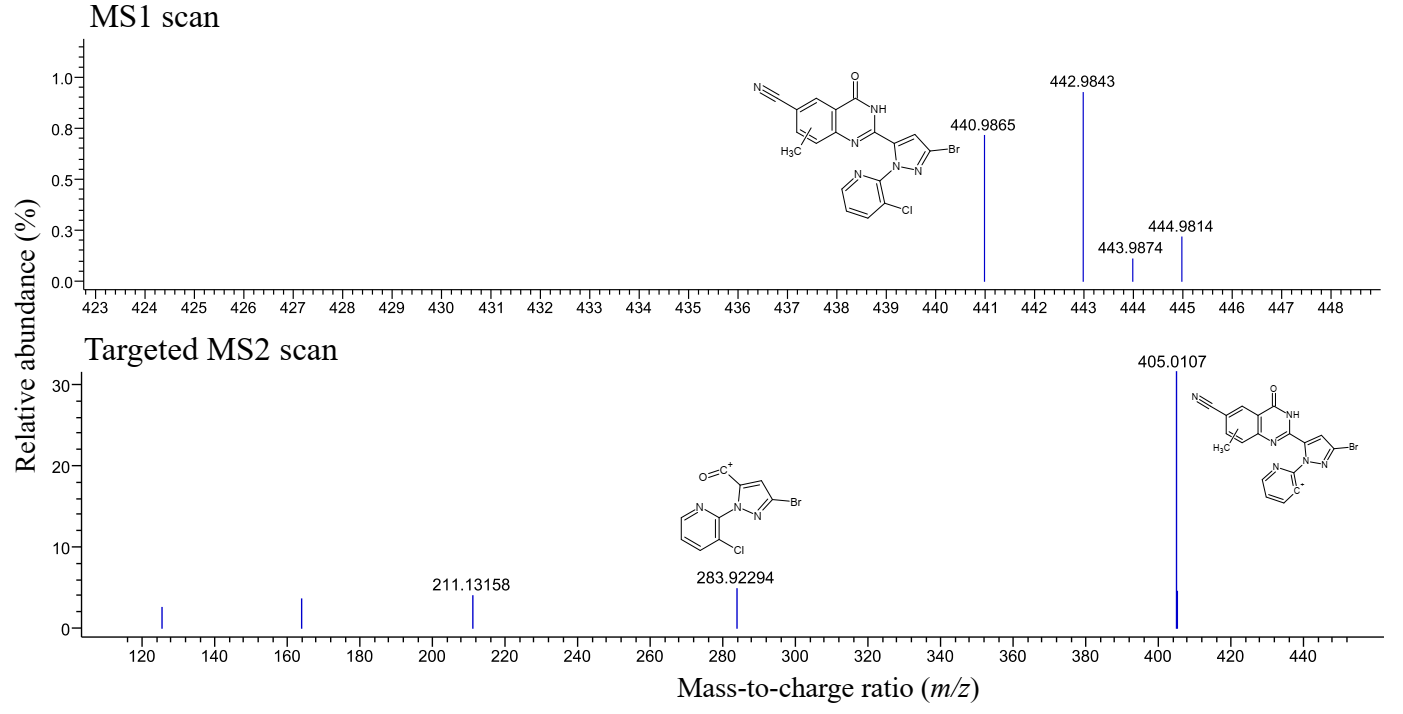 |
| --- |
| **Figure S14.** Mass spectra of TP441 on the UPLC-Orbitrap-MS system, acquired using CID mode (mass errors <4 ppm). |

| 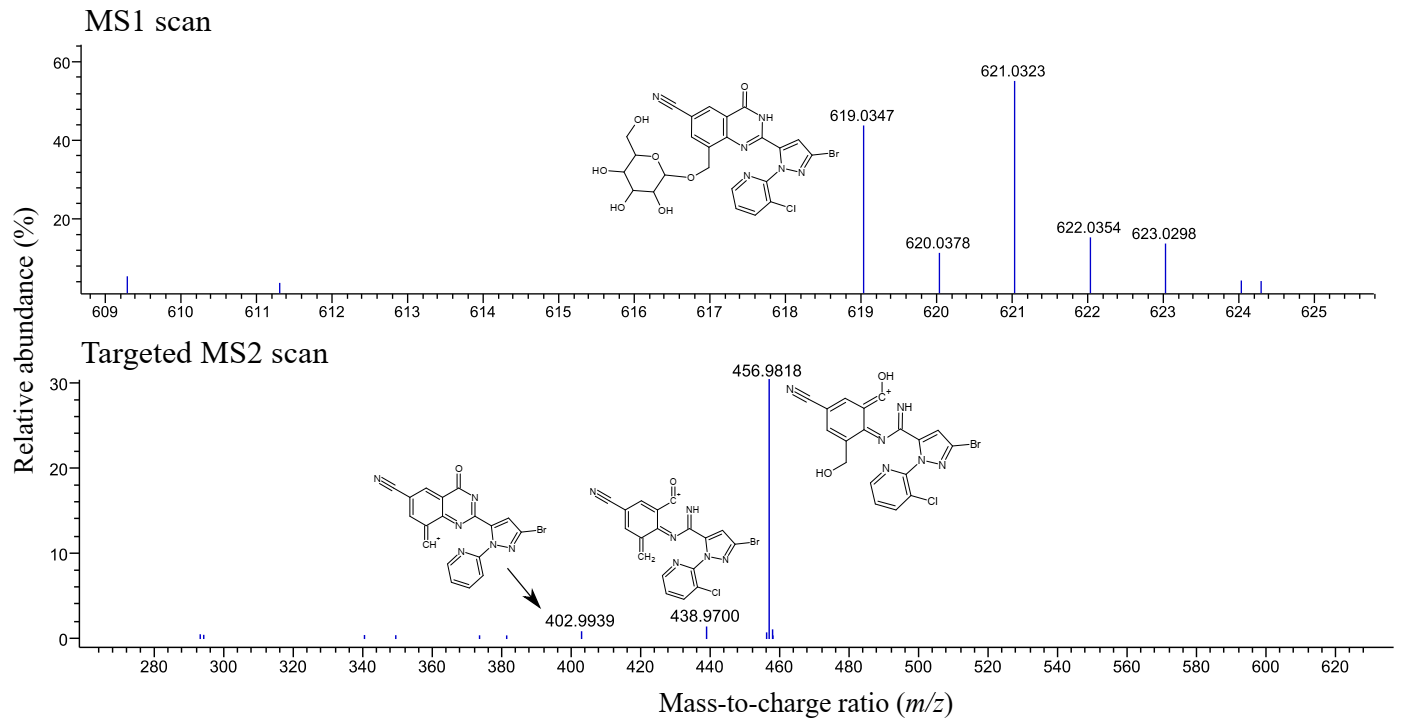 |
| --- |
| **Figure S15.** Mass spectra of TP619 on the UPLC-Orbitrap-MS system, acquired using CID mode (mass errors <2 ppm). |

| 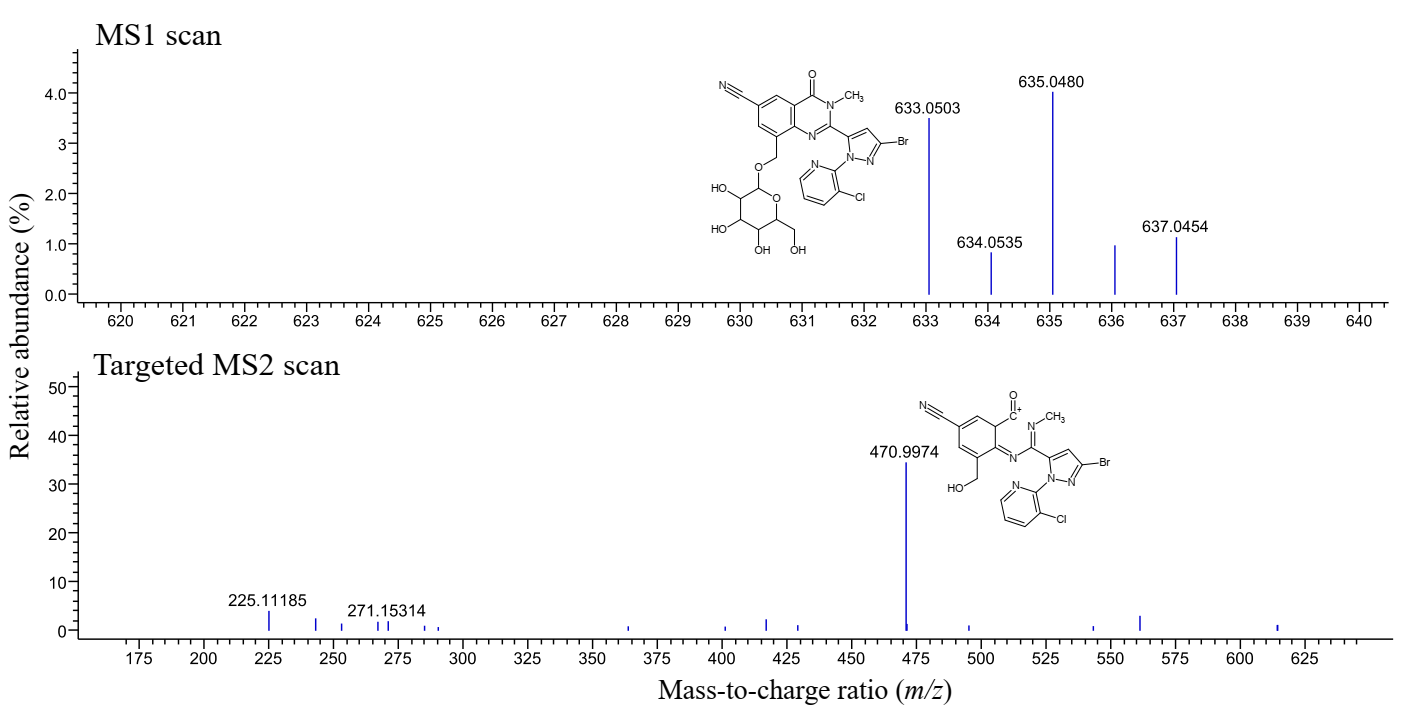 |
| --- |
| **Figure S16.** Mass spectra of TP633 on the UPLC-Orbitrap-MS system, acquired using CID mode (mass errors <2 ppm). |

| 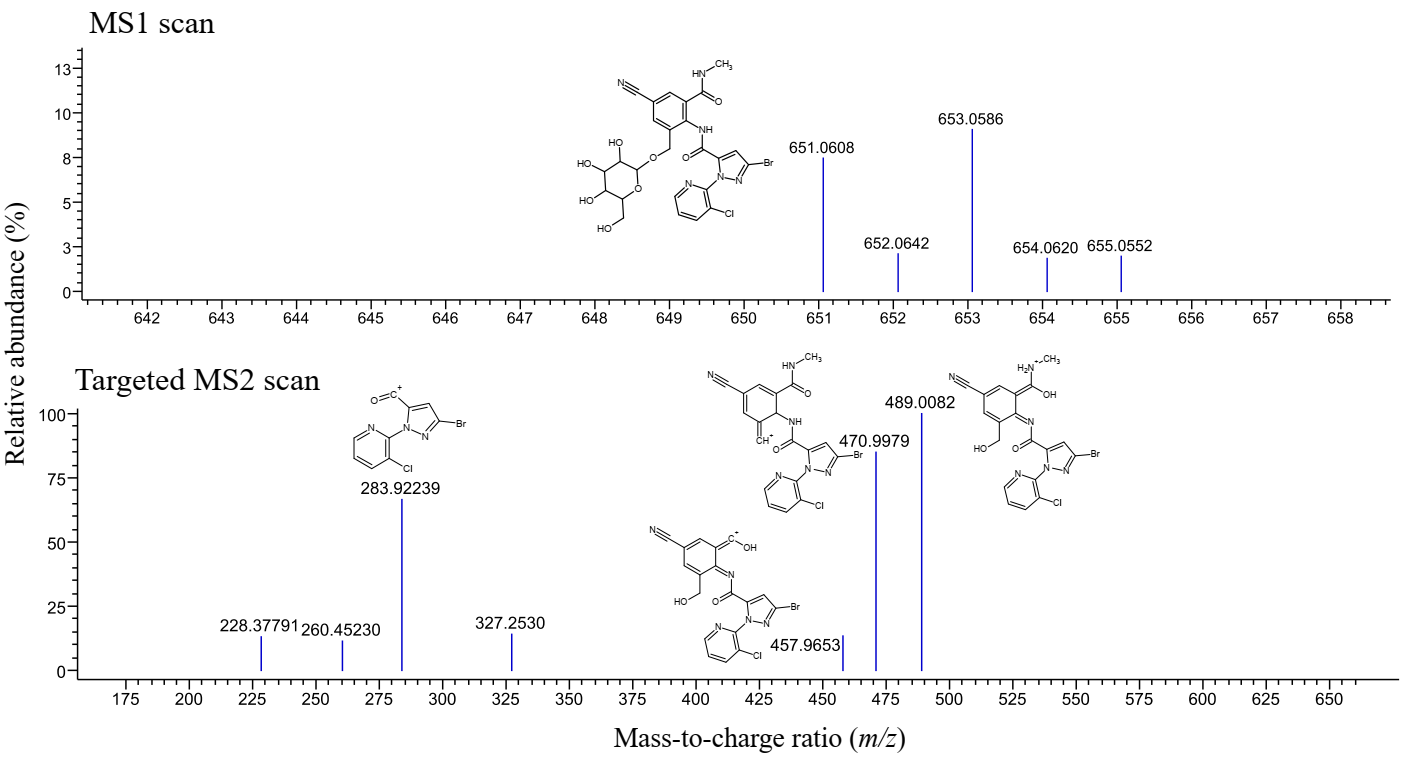 |
| --- |
| **Figure S17.** Mass spectra of TP651a on the UPLC-Orbitrap-MS system, acquired using CID mode (mass errors <3 ppm). |

| 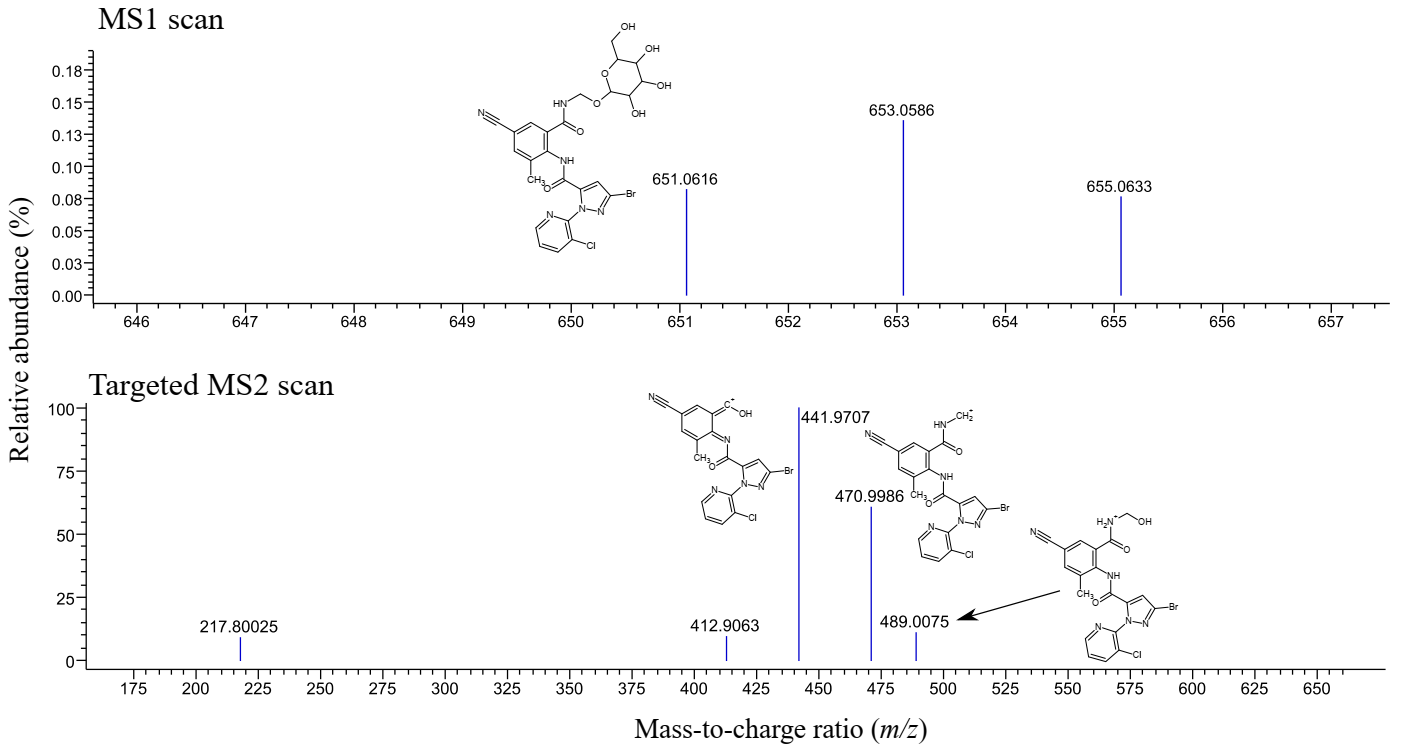 |
| --- |
| **Figure S18.** Mass spectra of TP651b on the UPLC-Orbitrap-MS system, acquired using CID mode (mass errors <5 ppm). |

| 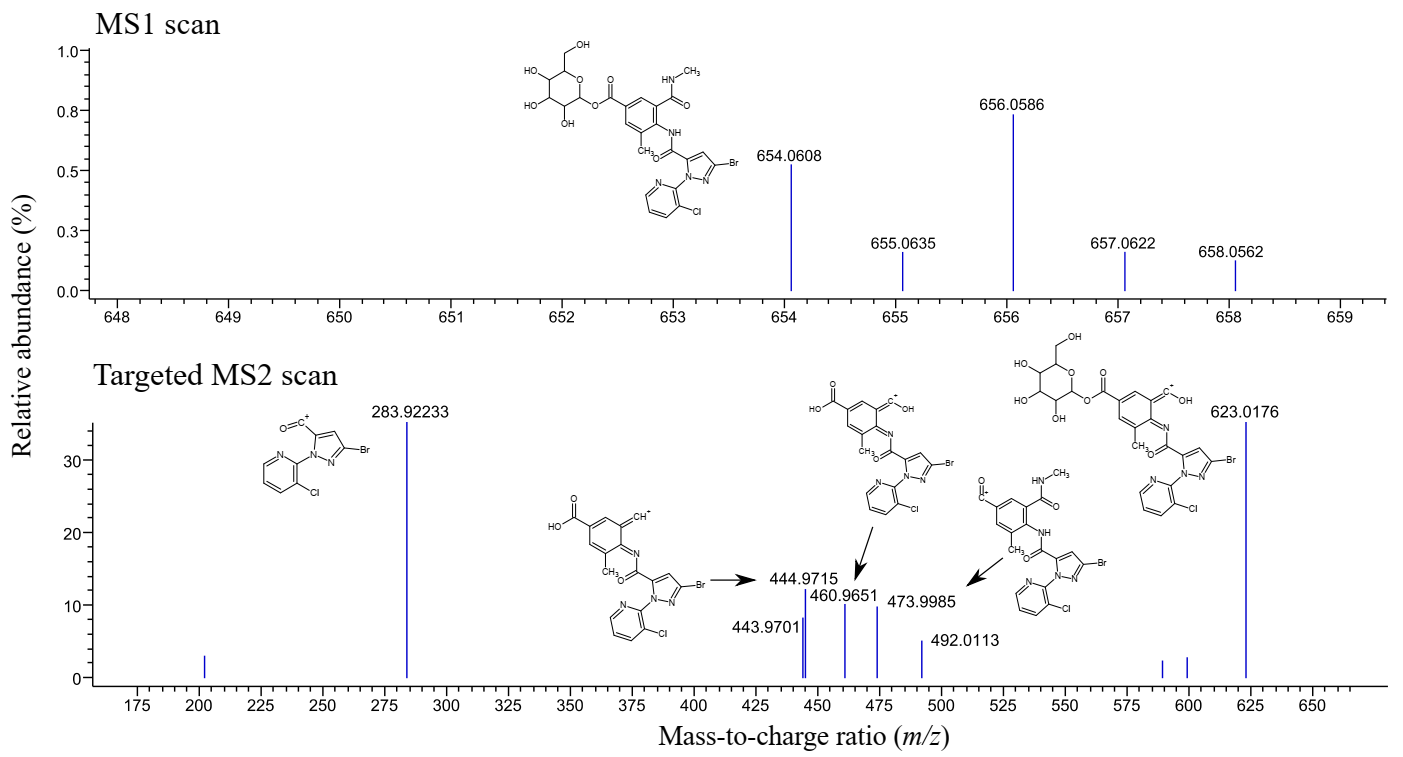 |
| --- |
| **Figure S19.** Mass spectra of TP654 on the UPLC-Orbitrap-MS system, acquired using CID mode (mass errors <5 ppm). |

| 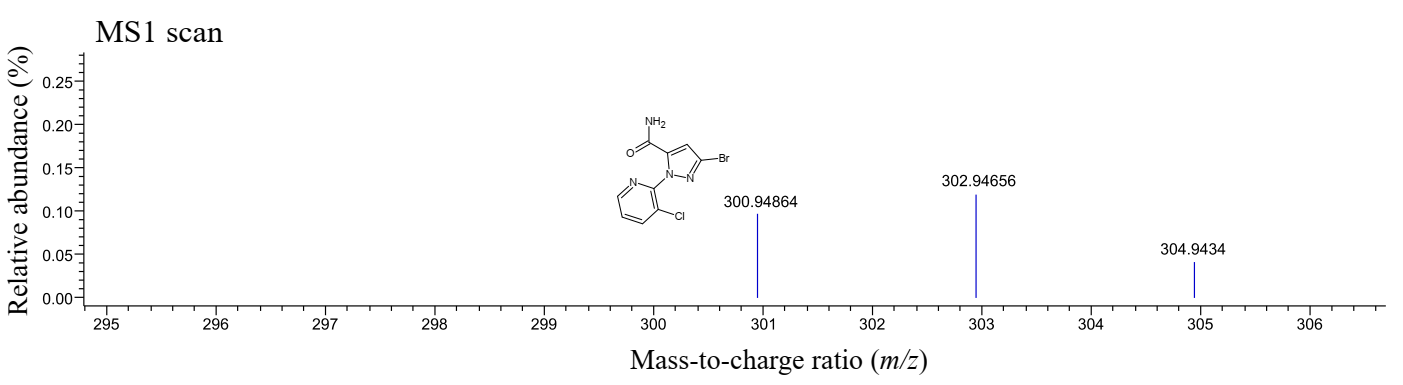 |
| --- |
| **Figure S20.** Full scan mass spectra of IN-M2G98 on the UPLC-Orbitrap-MS system (mass error = 0.0 ppm). |

| 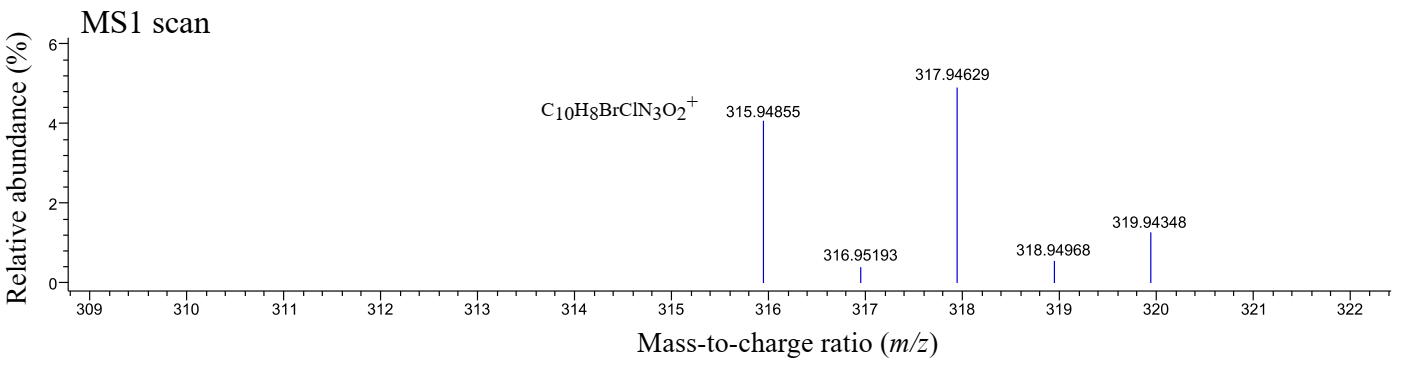 |
| --- |
| **Figure S21.** Full scan mass spectra of TP316 on the UPLC-Orbitrap-MS system (mass error = 1.0 ppm). |

| 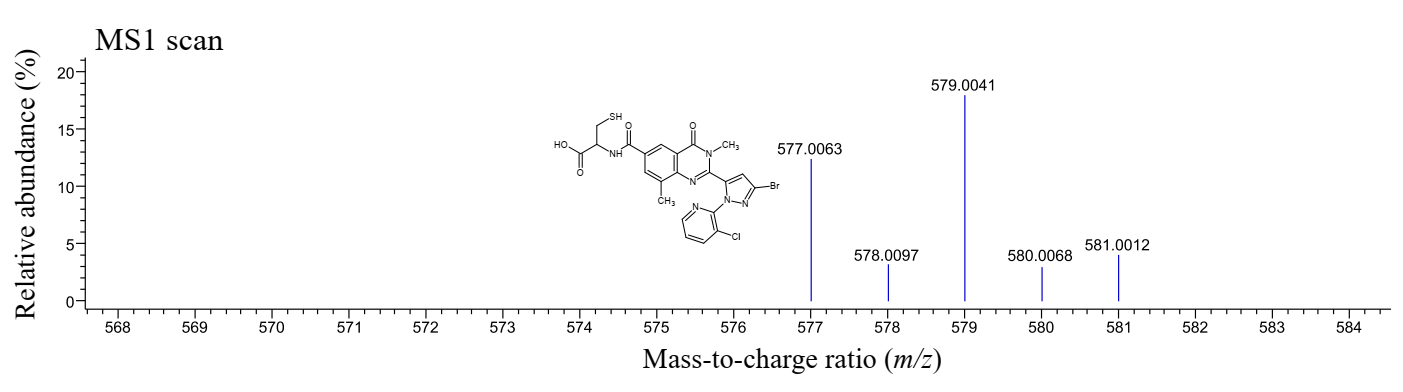 |
| --- |
| **Figure S22.** Full scan mass spectra of TP577 on the UPLC-Orbitrap-MS system (mass error = 1.4 ppm). |

| 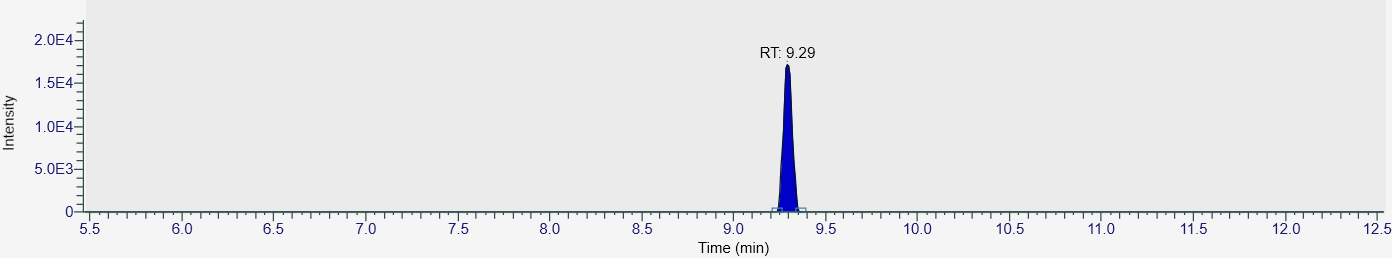  Red fruit matrix: 2 ng/mL  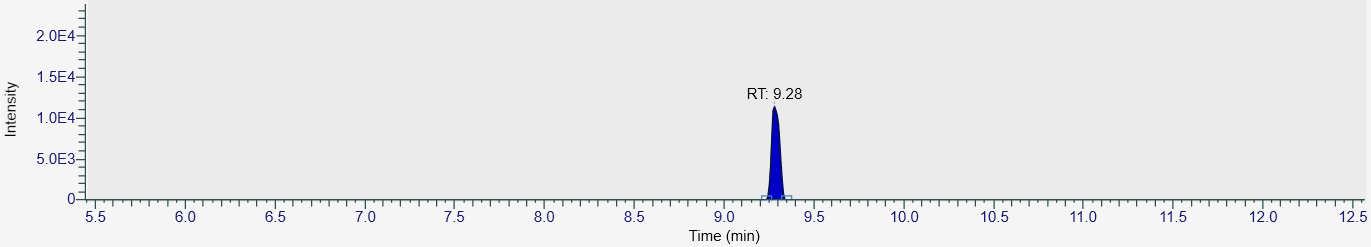  Green fruit matrix: 1 ng/mL  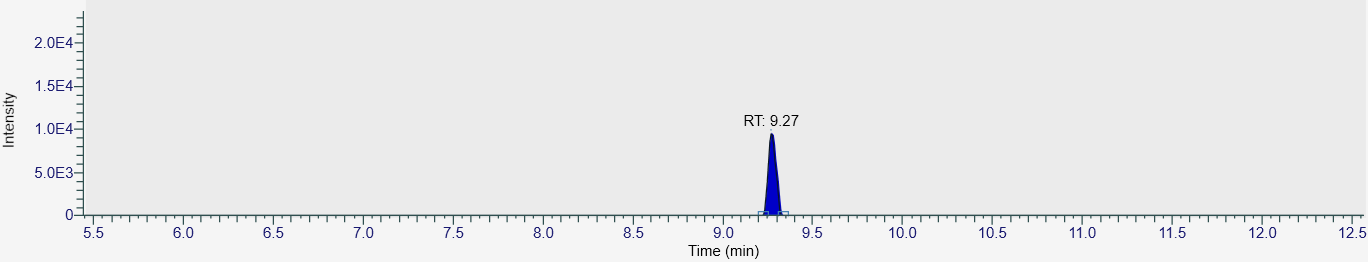  Young leaf matrix: 1 ng/mL  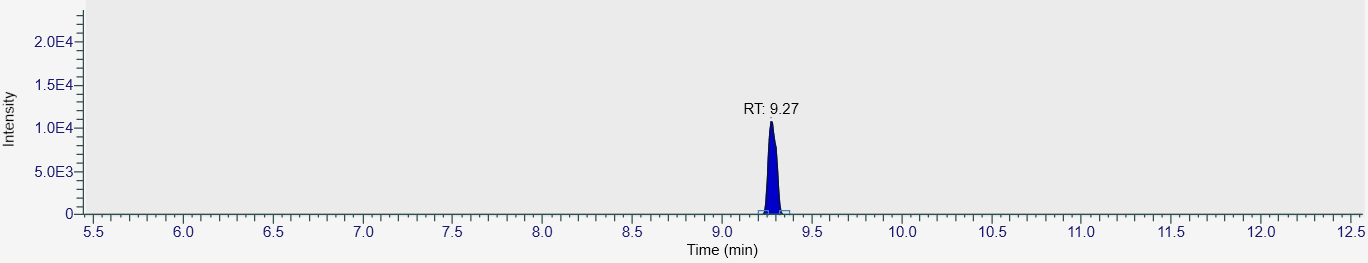  Mature leaf matrix: 1 ng/mL  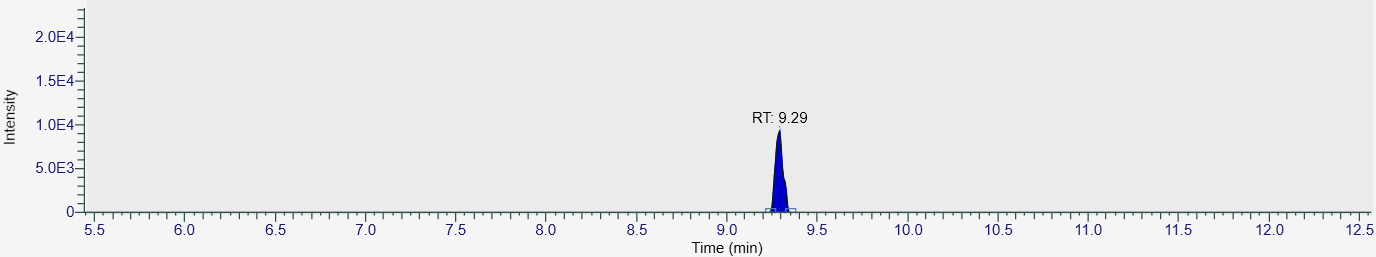  Flower matrix: 1 ng/mL  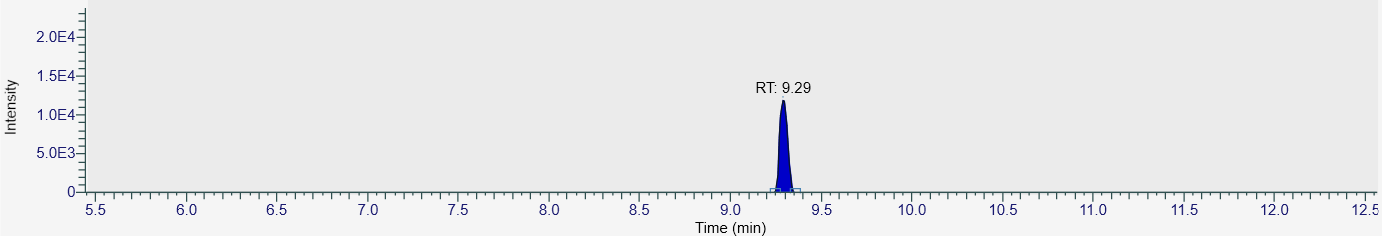  Acetonitrile: 0.8 ng/mL |
| --- |
| **Figure S23**. Chromatograms of cyantraniliprole in different sample matrices at the reported levels of quantification. |

| 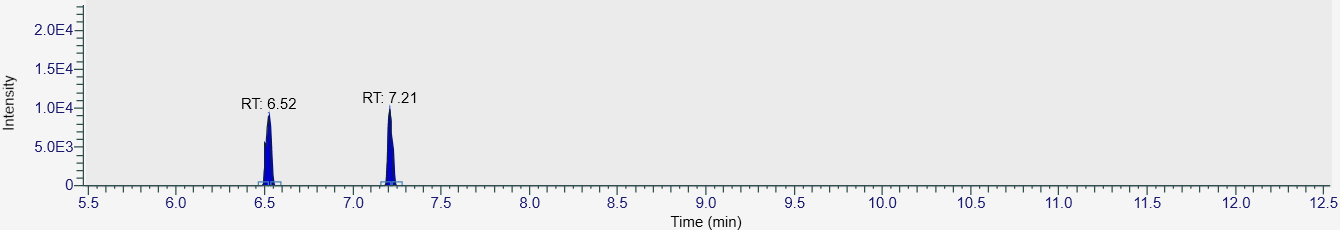  TP651  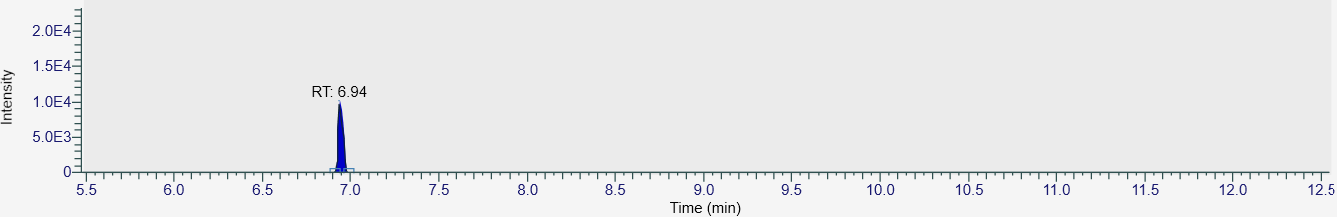  TP619  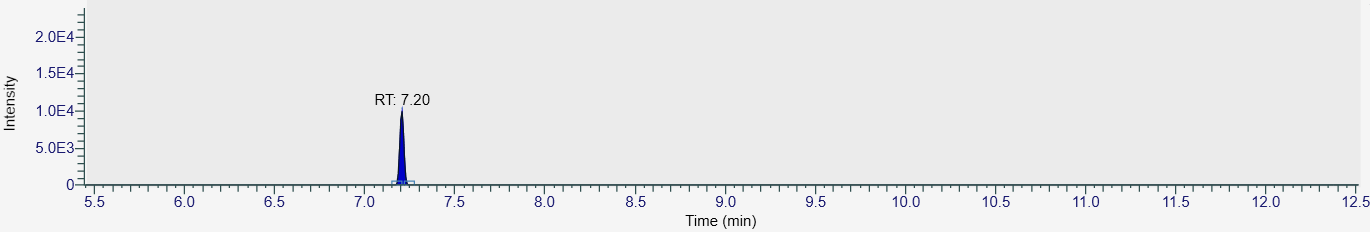  TP654  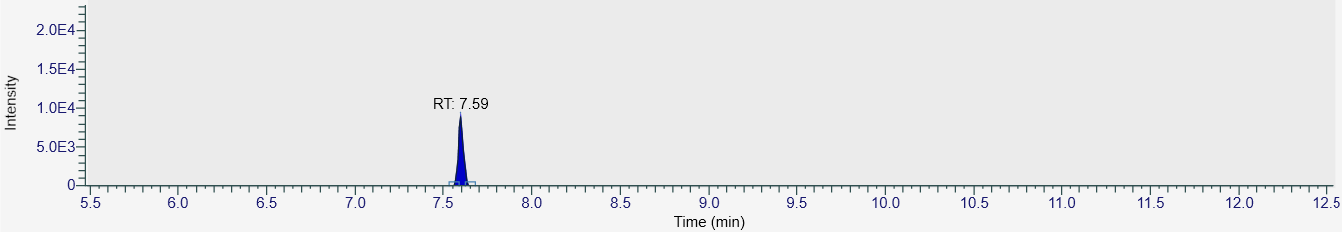  TP633  IN-JCZ38  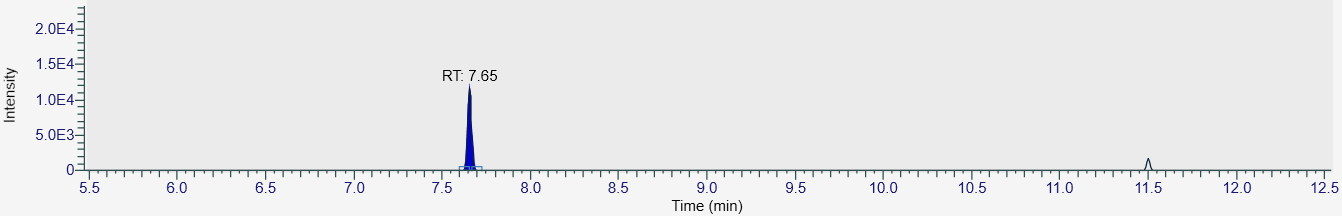  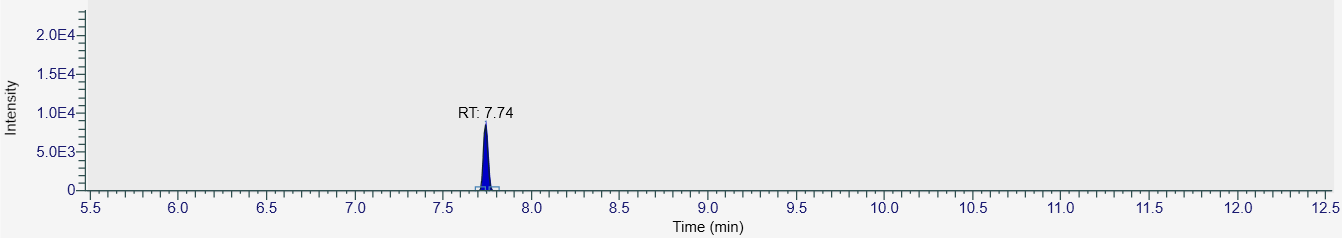  IN-DBC80  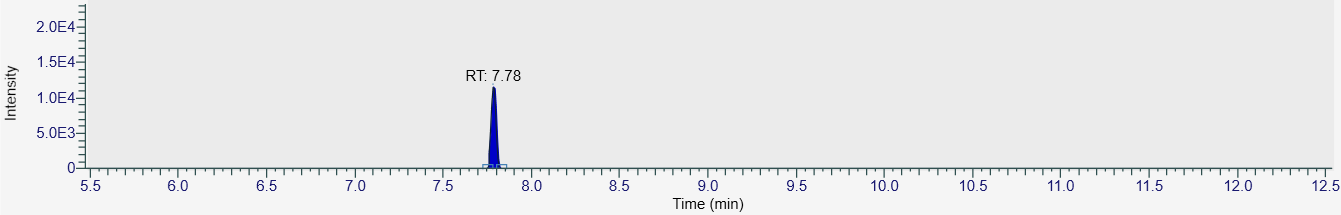  TP423  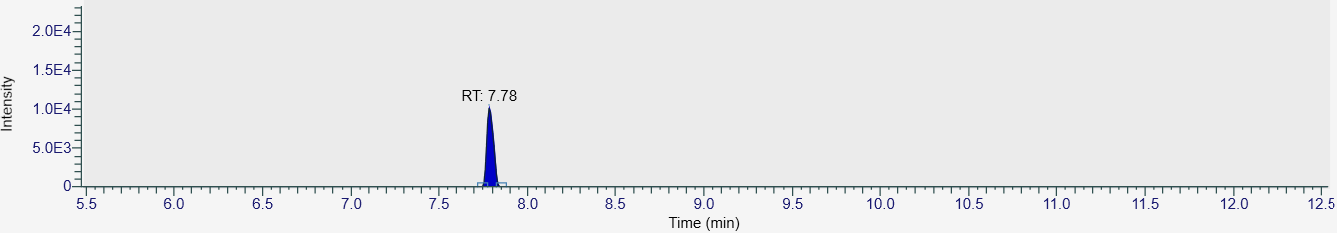  TP315  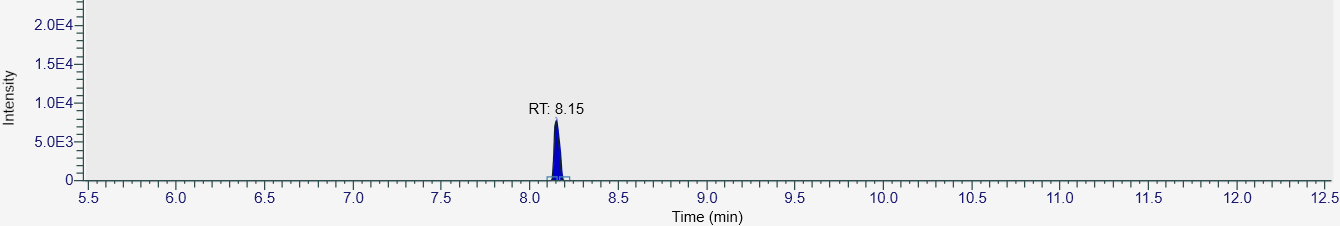  IN-RNU71  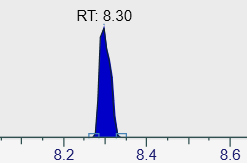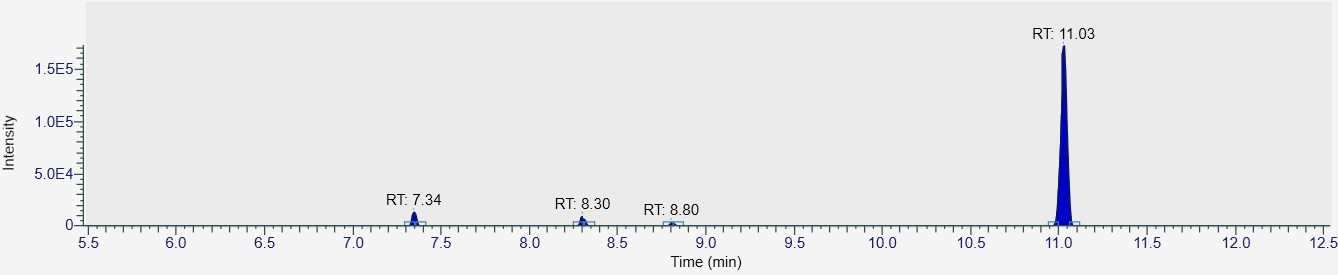  IN-HGW87  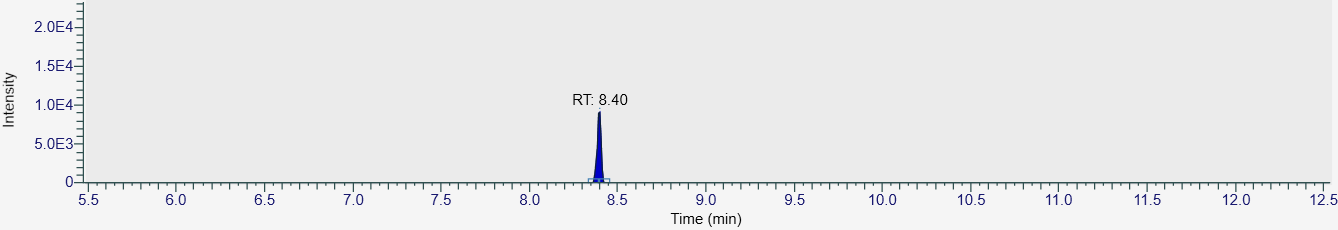  IN-JSE76  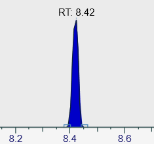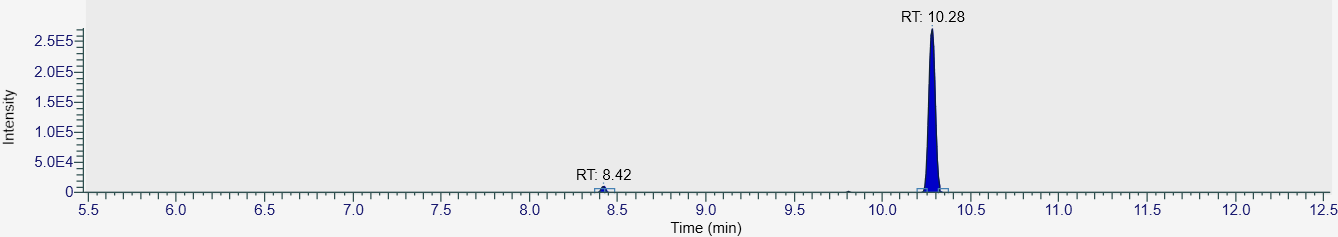  TP441  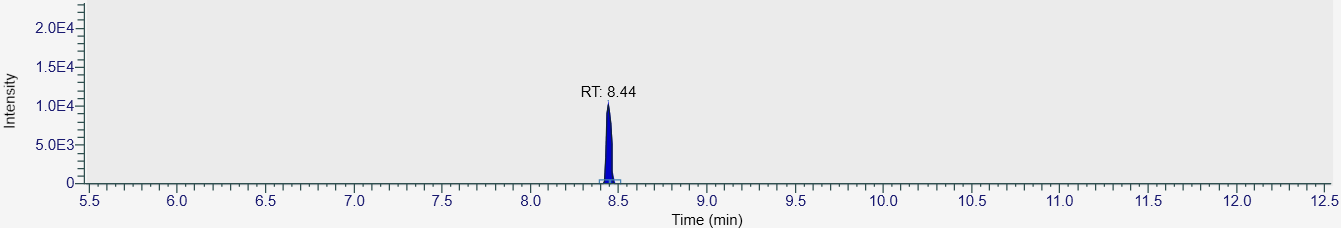  IN-MYX98  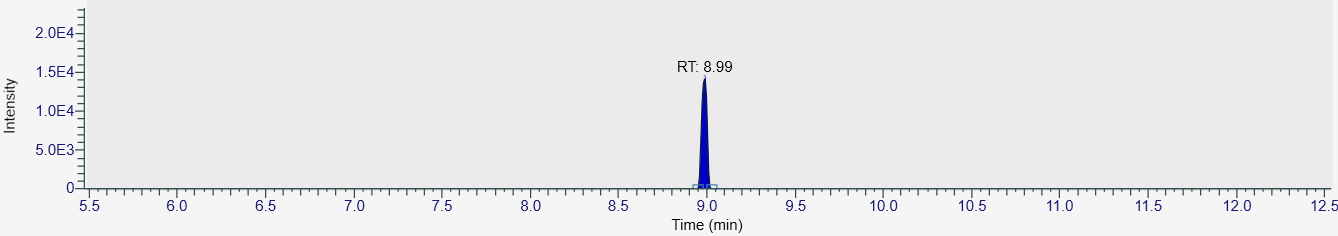  TP363  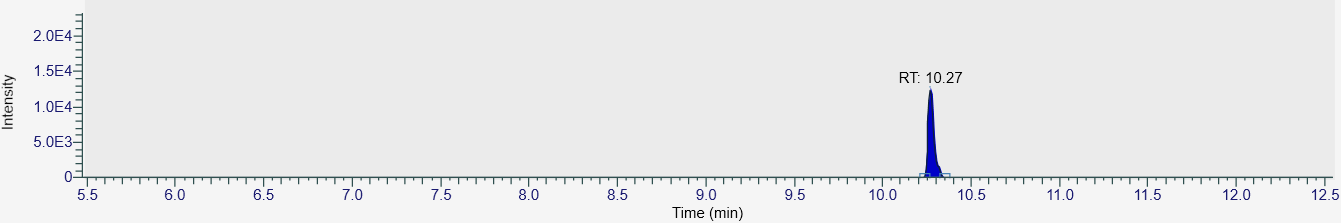  IN-MLA84  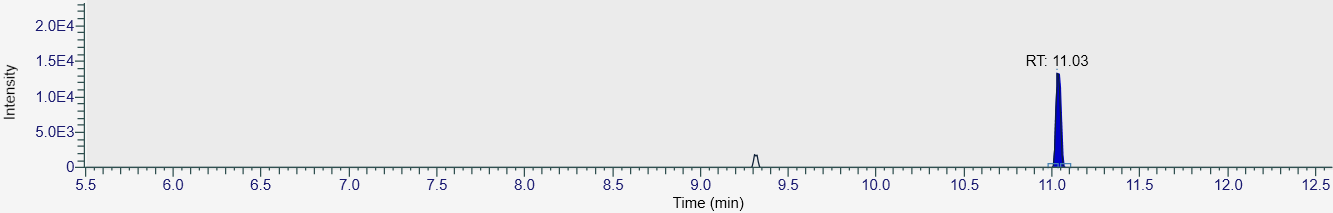  IN-J9Z38  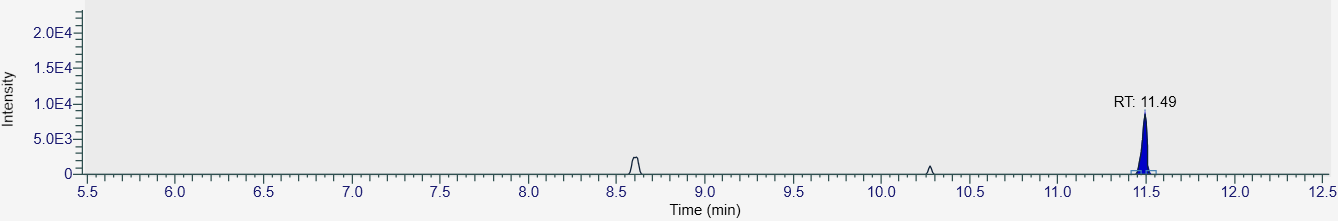  TP405 |
| --- |
| **Figure S24**. Chromatograms of cyantraniliprole metabolites at the levels of relative quantification reported in Figures 4 and S25. |

| 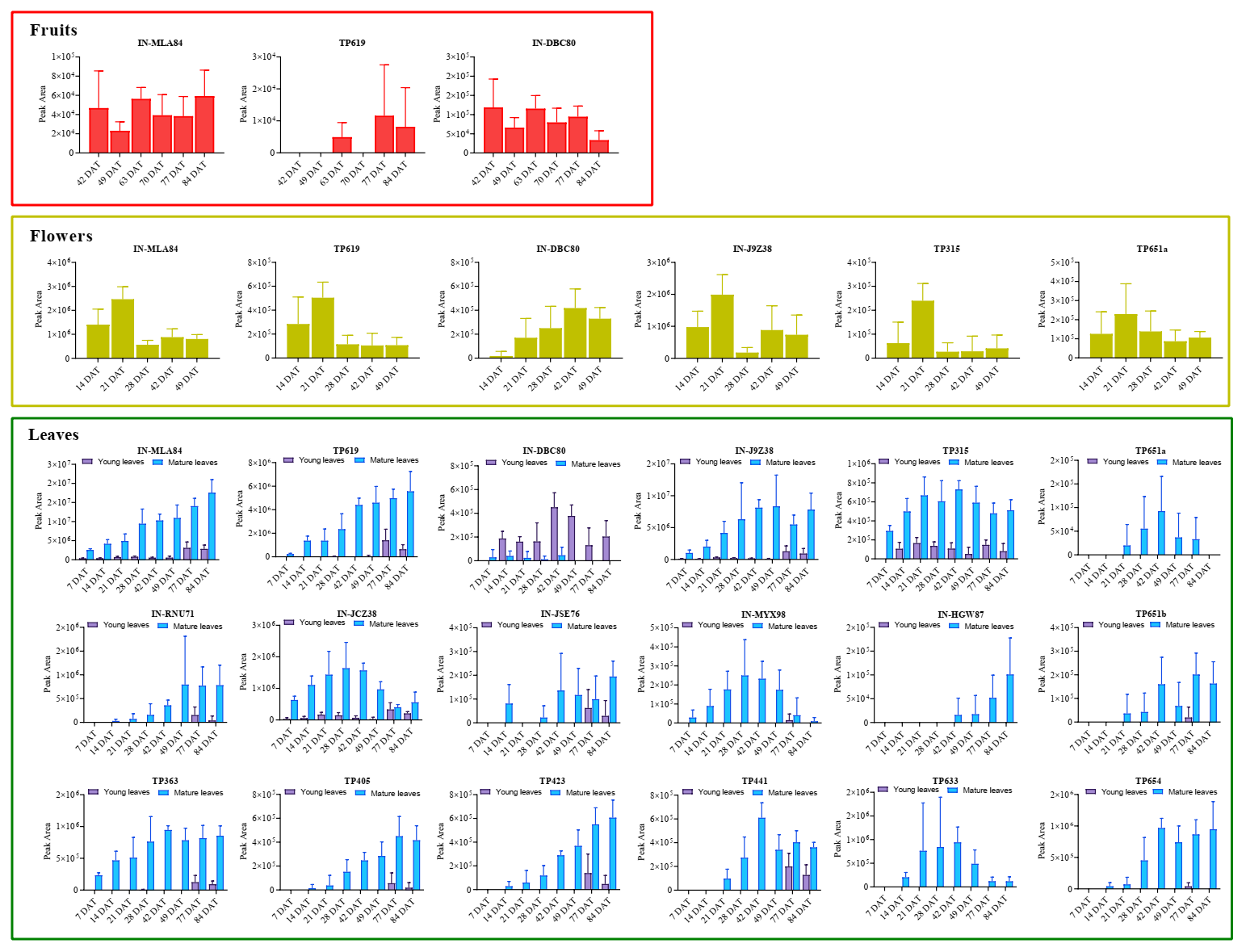 |
| --- |
| **Figure S25.** Normalized peak area (per one gram of fresh biomass) of cyantraniliprole metabolites detected in tomato leaves, flowers, and fruits throughout the experiments. For flowers, the metabolites that were only detectable in the concentrated extracts as described in the Materials and Methods, including IN-M2G98, IN-RNU71, IN-MYX98, IN-JCZ38, TP363, and TP423, are not presented due to their negligible abundance compared to parent cyantraniliprole. Also, other metabolites confirmed at the lowest confidence level (level 5, Table 1), including IN-M2G98, TP316, and TP577, are not presented for all plant tissues. The values represent the means ± standard deviation (*n* = 5). |

| **Table S1**. Sampling intervals scheduled for xylem sap and plant tissues. At 63 and 70 DAT, only fruit samples were harvested for cyantraniliprole and metabolites analysis. | | | | | | | | | | |
| --- | --- | --- | --- | --- | --- | --- | --- | --- | --- | --- |
|  | Days after cyantraniliprole application (DAT) | | | | | | | | | |
|  | 7 | 14 | 21 | 28 | 42 | 49 | 63 | 70 | 77 | 84 |
| Xylem sap | x | x | x | x | x | x |  |  | x | x |
| Leaves | x | x | x | x | x | x |  |  | x | x |
| Flowers |  | x | x | x | x | x |  |  |  |  |
| Fruits |  |  |  |  | x | x | x | x | x | x |

| **Table S2.** Limit of detection (LOD) and limit of quantification (LOQ) of cyantraniliprole in different plant matrices. | | | | |
| --- | --- | --- | --- | --- |
| Plant matrix | In plant extract (ng/mL) | | In fresh biomass (ng/g) | |
|  | LOD | LOQ | LOD | LOQ |
| Young leaves | 0.28 | 0.93 | 1.69 | 5.58 |
| Mature leaves | 0.31 | 1.03 | 1.88 | 6.19 |
| Flowers | 0.39 | 1.29 | 2.34 | 7.72 |
| Green fruits | 0.38 | 1.25 | 0.08 | 0.25 |
| Red fruits | 0.66 | 2.17 | 0.13 | 0.43 |
| Xylem sap (direct injection) | 0.34 | 1.13 |  |  |

| **Table S3.** List of cyantraniliprole metabolites in plants, compiled from the U.S. EPA’s registration documents EPA-HQ-OPP-2011-0668-0009^1^. | | | |
| --- | --- | --- | --- |
| **Metabolite** | **Formula** | **[M + H]^+^** | **Structure** |
| IN-DBC80 | C_9_H_5_BrClN_3_O_2_ | 301.9326 |  |
| IN-J9Z38 | C_19_H_12_BrClN_6_O | 455.0017 |  |
| IN-NBC94 | C_19_H_12_BrClN_6_O_2_ | 470.9966 |  |
| IN-JCZ38 | C_19_H_16_BrClN_6_O_3_ | 491.0228 |  |
| IN-JSE76 | C_19_H_15_BrClN_5_O_4_ | 492.0069 |  |
| IN-K5A77 | C_19_H_14_BrClN_6_O_2_ | 473.0123 |  |
| IN-K5A78 | C_19_H_13_BrClN_5_O_3_ | 473.9963 |  |
| IN-K5A79 | C_18_H_13_BrClN_5_O_4_ | 477.9912 |  |
| IN-K7H19 | C_18_H_14_BrClN_6_O_3_ | 477.0072 |  |
| IN-MLA84 | C_18_H_10_BrClN_6_O | 440.9861 |  |
| Hydroxyl IN-MLA84 | C_18_H_10_BrClN_6_O_2_ | 456.9810 |  |
| Bis-hydroxy cyantraniliprole | C_19_H_14_BrClN_6_O_4_ | 505.0021 |  |
| IN-MYX98 | C_19_H_14_BrClN_6_O_3_ | 489.0072 |  |
| IN-N7B69 | C_19_H_14_BrClN_6_O_3_ | 489.0072 |  |
| IN-N7B69 Glucoside | C_25_H_24_BrClN_6_O_8_ | 651.0600 |  |
| IN-NXX70 | C_19_H_13_BrN_6_O_2_ | 437.0356 |  |
| IN-QKV54 | C_14_H_10_BrN_5_O | 344.0141 |  |
| IN-HGW87 | C_18_H_12_BrClN_6_O_2_ | 458.9966 |  |

References

1. USEPA. Cyantraniliprole. Petition for the Establishment of Permanent Tolerances and Registration for Use on Brassica (cole) Leafy Vegetables (Group 5), Bulb Vegetables (Group 3-07), Bushberries (Group 13-07B), Oilseed (Group 20), Citrus Fruit (Group 10-10), Cucurbit Vegetables (Group 9), Fruiting Vegetables (Group 8-10), Leafy Vegetables (non-Brassica) (Group 4), Pome Fruit (Group 11-10), Stone Fruit (Group 12), Tree Nuts (Group 14), Tuberous and Corm Vegetables (Subgroup 1C), and Food/Feed Handling Establishments. Summary of Analytical Chemistry and Residue Data. Report No. EPA-HQ-OPP-2011-0668-0009, (Office of Chemical Safety and Pollution Prevention, Washington DC, 2013).
